# Supplementary material for: Enzyme Replacement Therapy Can Reverse Pathogenic Cascade in Pompe Disease
Source: Mol Ther Methods Clin Dev. 2020 Jun 10;18:199–214. doi: 10.1016/j.omtm.2020.05.026 (PMC7334420; doi:10.1016/j.omtm.2020.05.026)
Supplement: Document S1. Figures S1–S3, and Table S1 [file mmc1.pdf]

OMTM, Volume *18*

Supplemental Information

Enzyme Replacement Therapy Can Reverse Pathogenic Cascade in Pompe Disease

Naresh Kumar Meena, Evelyn Ralston, Nina Raben, and Rosa Puertollano

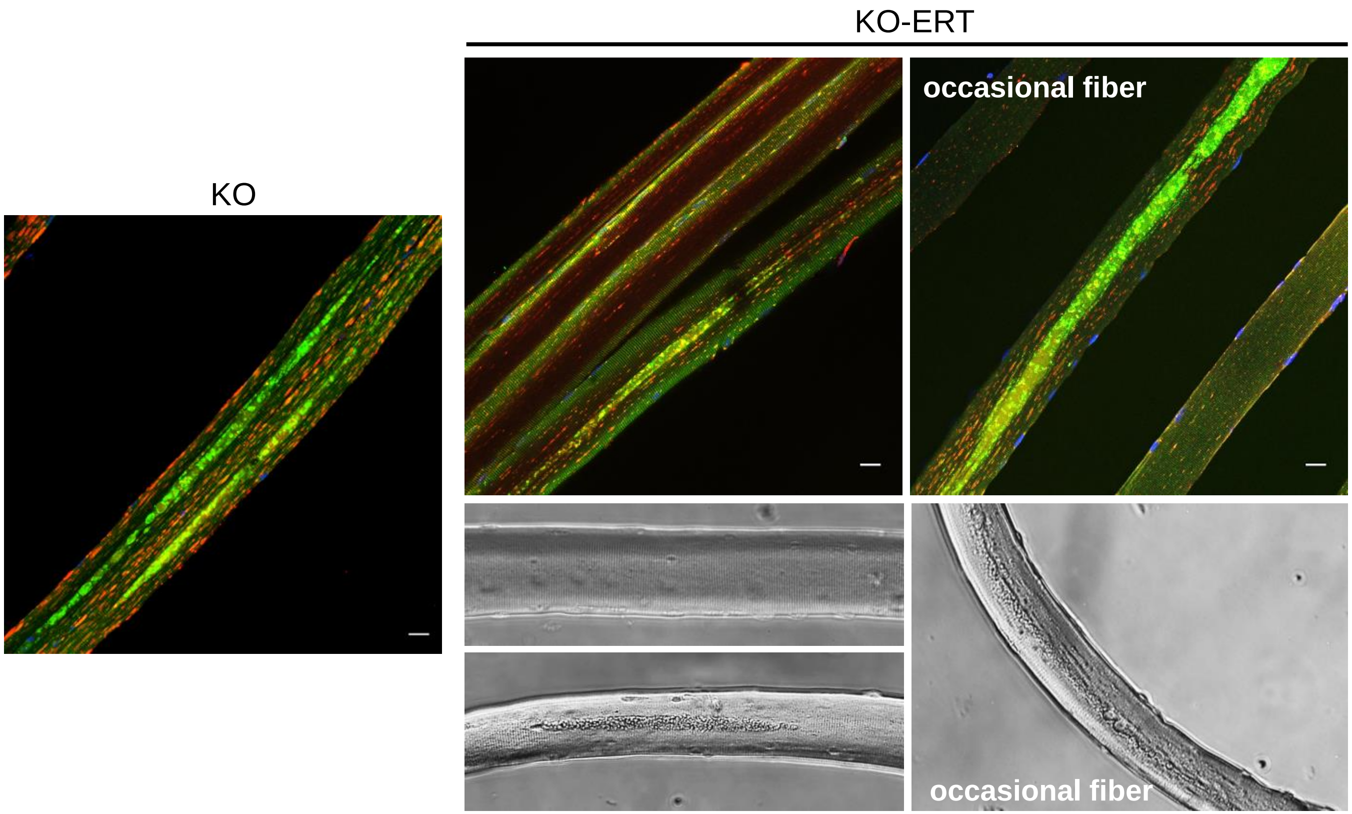

Figure S1. Additional images of muscle fibers from untreated- and treated KO mice.

Confocal microscopy images of single muscle fibers immunostained with lysosomal marker LAMP1 (red) and autophagosomal marker LC3 (green); nuclei are stained with Hoechst dye (blue). Virtually all muscle fibers (the white part of gastrocnemius) from 9-month-old untreated KO contain large areas of autophagic buildup often spanning the whole length of the fibers. Most fibers from 9-month-old treated KO mice (the animals received 11 bi-weekly i.v. administrations of AT-GAA as described in the main text) are free from autophagic buildup, whereas ~16% fibers contain autophagic buildup of smaller size, and only occasional fibers contain autophagic buildup of the size as the one seen in untreated KO mice. Autophagic buildup can also be detected by bright-field microscopy or differential interference contrast microscopy. Bar: 20 µm.

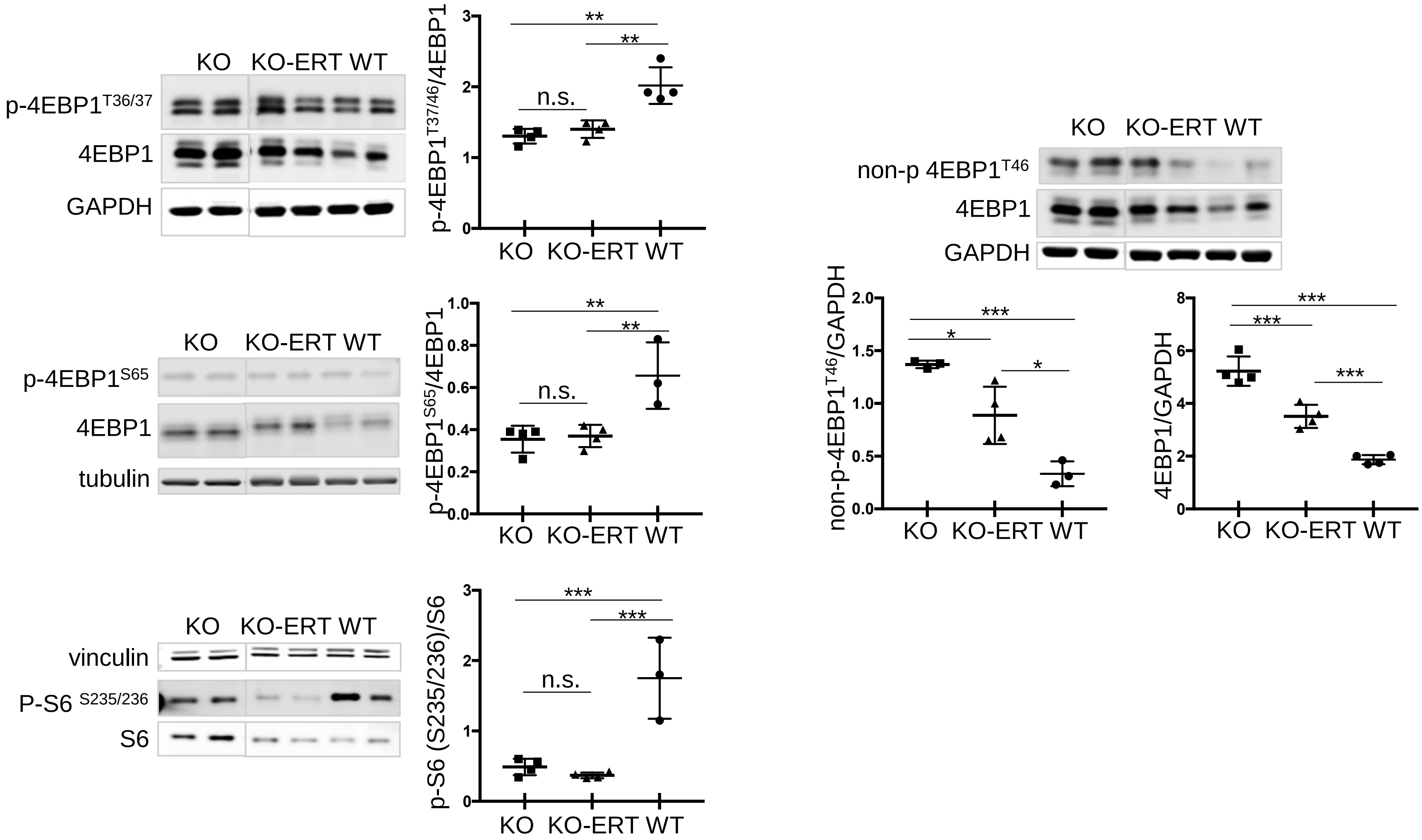

Figure S2. The effect of AT-GAA on mTORC1 signaling following a short-term regimen. KO mice (3-4- month-old) received 4 bi-weekly i.v. administrations of AT-GAA as described in the main text. Age and sex-matched WT and untreated KO mice were used for the comparisons. Skeletal muscle biopsies (the white part of gastrocnemius) were collected 14 days after the last administration. Western blot analysis of muscle lysates from WT, untreated KO (KO), and treated KO (KO-ERT) mice with indicated antibodies (n=3-4 for each group). Different forms of 4EBP1, phosphorylated (p-4EBP1$^{T37/46}$ and p-4EBP1^{S65}), non-phosphorylated (non-p-4EBP1^{T46}) and total were analyzed. Similar to what was found after 11 i.v. administrations of AT-GAA, no changes in the mTOR activity are detected in treated compared to untreated KO samples. Again, similar to what was found after 11 i.v. administrations of AT-GAA, a decrease in both non-p-4EBP1^{T46} and total is seen in treated compared to untreated KO muscle GAPDH and vinculin were used as loading controls. Each data point represents an individual mouse. Statistical significance was determined by one-way ANOVA. Graphs represent mean ± SD. $^{*}P<0.05$; ** $P<0.01$; *** $P<0.001$.

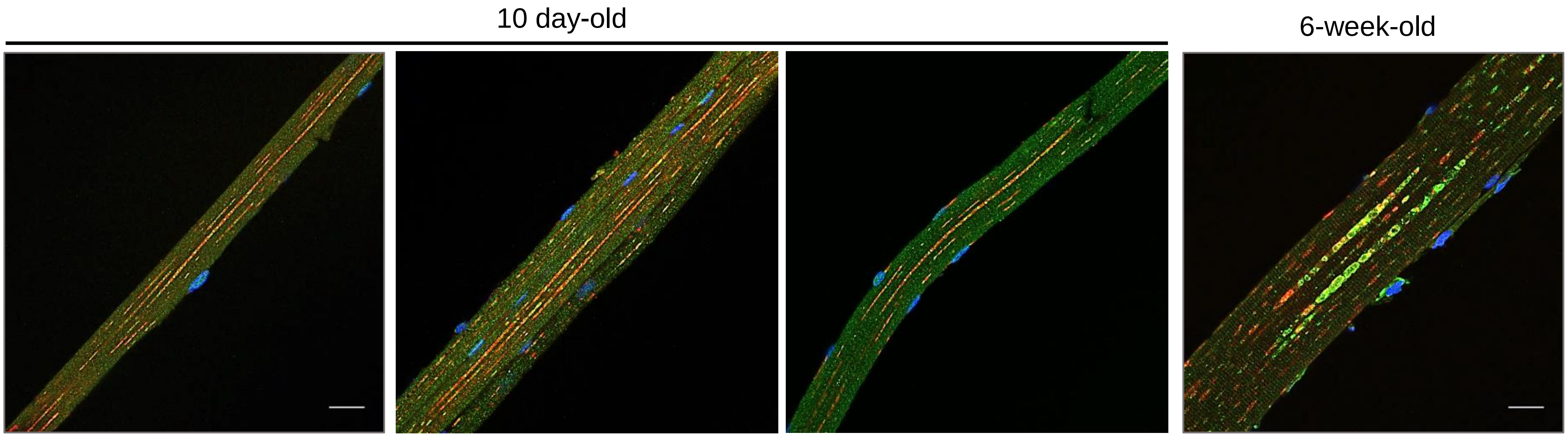

Figure S3. Autophagic buildup in KO muscle can be detected as early as in 10-day-old animals. Confocal microscopy images of single muscle fibers immunostained with lysosomal marker LAMP1 (red) and autophagosomal marker LC3 (green); nuclei are stained with Hoechst dye (blue). The fibers are isolated from muscle (the white part of gastrocnemius) derived from 10-day-old and 6-week-old mice. Bar: 20 μm.

Video S1. Confocal microscopy images of serial optical sections taken from two adjacent KO muscle fibers stained with lysosomal marker LAMP1 (red)and autophagosomal marker LC3 (green). The images show the extent of the autophagic buildup in fibers derived from untreated KO mice. Note, that the buildup in the left fiber surfaces before that in the right fiber, indicating that these structures belong to different focal planes along the Z stack. It is therefore clear that the autophagic buildup can be easily missed on routine histology of longitudinal sections of the diseased skeletal muscle.

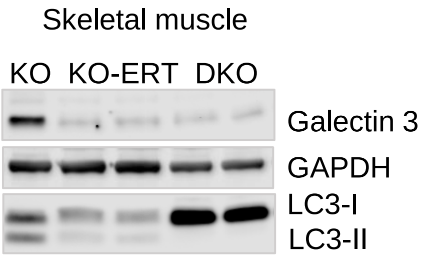

Raw data for Figure 5C

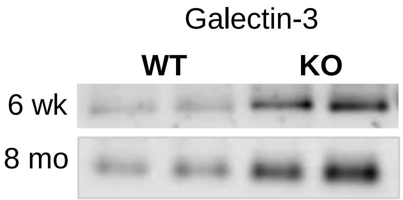

(referred to as “not shown” in the main text)

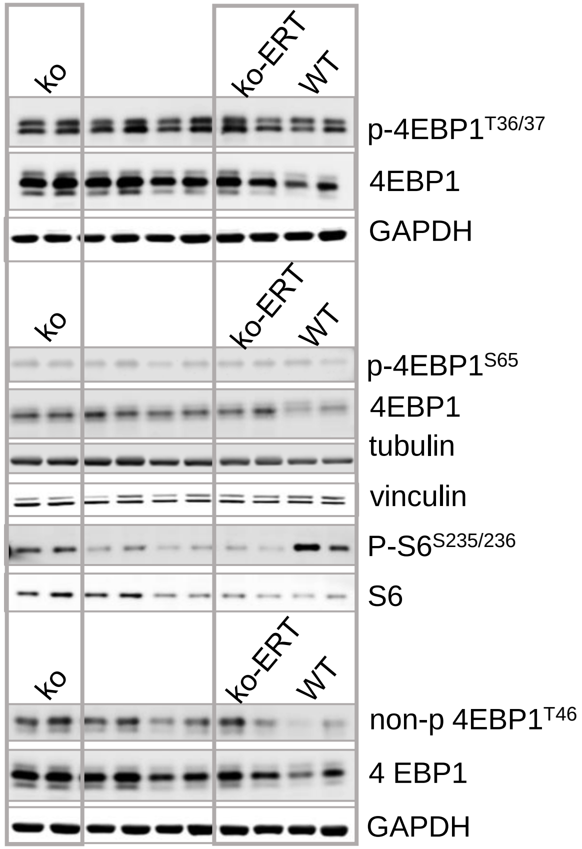

Raw data for Supplemental Figure S2

Supplemental Table 1: Absolute Concentration of 116 Compounds

	HMT DB	Concentration (nmol/g)						Comparative Analysis					
ID	Compound name	KO-Treatment		KO		WT		KO-Treatment vs KO			KO vs WT		
		Mean	S.D.	Mean	S.D.	Mean	S.D.	Ratio ¶	*p*-value ‖		Ratio ¶	*p*-value ‖	
A_0001	NAD^{+}	479	74	548	19	485	61	0.9	0.071		1.1	0.052	
A_0002	cAMP	0.9	0.3	1.0	0.2	0.9	0.2	0.9	0.676		1.1	0.621	
A_0003	cGMP	N.A.	N.A.	0.02	0.002	N.A.	N.A.	<1	N.A.		1<	N.A.	
A_0004	NADH	9.6	0.7	11	1.5	9.4	1.0	0.9	0.223		1.1	0.147	
A_0005	Xanthine	1.1	0.3	1.8	1.0	0.4	0.3	0.6	0.159		4.2	0.021	*
A_0006	ADP-ribose	8.1	5.9	8.9	2.6	5.9	2.5	0.9	0.780		1.5	0.069	
A_0007	Mevalonic acid	N.A.	N.A.	N.A.	N.A.	N.A.	N.A.	N.A.	N.A.		N.A.	N.A.	
A_0008	UDP-glucose	18	5.7	24	1.2	15	6.6	0.8	0.052		1.6	0.023	*
A_0009	Uric acid	2.9	0.7	4.7	1.8	2.5	1.2	0.6	0.065		1.9	0.036	*
A_0010	NADP^{+}	12	2.1	17	1.3	11	1.3	0.7	0.001	**	1.6	7.0E-06	***
A_0011	IMP	78	20	98	32	158	79	0.8	0.235		0.6	0.131	
A_0012	Sedoheptulose 7-phosphate	N.A.	N.A.	N.A.	N.A.	N.A.	N.A.	N.A.	N.A.		N.A.	N.A.	
A_0013	Glucose 6-phosphate	1,427	372	1,343	217	1,765	271	1.1	0.643		0.8	0.015	*
A_0014	Fructose 6-phosphate	623	157	528	93	777	106	1.2	0.241		0.7	0.002	**
A_0015	Fructose 1-phosphate	N.A.	N.A.	N.A.	N.A.	N.A.	N.A.	N.A.	N.A.		N.A.	N.A.	
A_0016	Galactose 1-phosphate	5.6	1.3	8.2	2.7	2.9	1.1	0.7	0.070		2.8	0.003	**
A_0017	Glucose 1-phosphate	166	70	146	27	228	54	1.1	0.526		0.6	0.011	*
A_0018	Acetoacetyl CoA	N.A.	N.A.	N.A.	N.A.	N.A.	N.A.	N.A.	N.A.		N.A.	N.A.	
A_0019	Acetyl CoA	0.4	0.2	0.6	0.2	0.4	0.14	0.7	0.085		1.7	0.033	*
A_0020	Folic acid	N.A.	N.A.	N.A.	N.A.	N.A.	N.A.	N.A.	N.A.		N.A.	N.A.	
A_0021	Ribose 5-phosphate	2.6	1.7	4.3	1.7	2.4	0.9	0.6	0.109		1.8	0.041	*
A_0022	CoA	1.8	0.7	2.2	0.6	2.7	1.2	0.8	0.305		0.8	0.351	
A_0023	Ribose 1-phosphate	4.7	0.9	7.6	2.5	3.5	2.1	0.6	0.033	*	2.2	0.012	*
A_0024	Ribulose 5-phosphate	8.3	5.3	10	2.1	7.7	5.4	0.8	0.439		1.3	0.318	
A_0025	Xylulose 5-phosphate	N.A.	N.A.	N.A.	N.A.	N.A.	N.A.	N.A.	N.A.		N.A.	N.A.	
A_0026	Erythrose 4-phosphate	N.A.	N.A.	N.A.	N.A.	N.A.	N.A.	N.A.	N.A.		N.A.	N.A.	
A_0027	HMG CoA	0.3	0.008	0.4	0.04	0.3	0.006	0.9	0.308		1.1	0.615	
A_0028	Glyceraldehyde 3-phosphate	4.2	3.5	13	7.6	5.2	6.7	0.3	0.038	*	2.5	0.089	
A_0029	NADPH	5.8	0.2	5.9	0.4	6.3	0.3	1.0	0.611		0.9	0.069	
A_0030	Malonyl CoA	0.5	0.2	0.7	0.13	0.5	0.05	0.7	0.102		1.4	0.012	*
A_0031	Phosphocreatine	2,234	331	2,182	142	1,997	361	1.0	0.738		1.1	0.282	
A_0032	XMP	0.12	0.02	0.3	0.04	0.11	0.02	0.4	3.4E-05	***	3.0	1.7E-05	***
A_0033	Dihydroxyacetone phosphate	153	68	254	67	246	109	0.6	0.027	*	1.0	0.877	
A_0034	Adenylosuccinic acid	1.9	0.4	2.8	1.1	2.2	1.2	0.7	0.113		1.2	0.429	
A_0035	Fructose 1,6-diphosphate	353	205	469	110	635	482	0.8	0.258		0.7	0.443	
A_0036	6-Phosphogluconic acid	2.4	0.5	2.9	0.4	3.6	1.7	0.8	0.093		0.8	0.387	
A_0037	*N*-Carbamoylaspartic acid	0.07	0.03	0.11	0.03	0.10	0.04	0.6	0.018	*	1.2	0.451	
A_0038	PRPP	17	5.0	19	6.7	23	9.4	0.9	0.631		0.8	0.351	
A_0039	2-Phosphoglyceric acid	3.3	0.8	3.0	0.6	2.9	0.9	1.1	0.510		1.0	0.851	
A_0040	2,3-Diphosphoglyceric acid	1.1	0.2	2.2	1.0	0.8	0.07	0.5	0.033	*	2.7	0.018	*
A_0041	3-Phosphoglyceric acid	33	5.7	30	5.9	29	6.7	1.1	0.293		1.0	0.760	
A_0042	Phosphoenolpyruvic acid	6.9	1.0	2.8	1.6	4.4	1.3	2.5	7.1E-04	***	0.6	0.083	
A_0043	GMP	1.6	0.3	2.3	0.14	1.5	0.4	0.7	0.001	**	1.6	0.003	**
A_0044	AMP	4.9	1.2	5.8	2.6	4.3	2.2	0.8	0.463		1.4	0.294	
A_0045	2-Oxoisovaleric acid	4.9	1.2	5.0	0.5	7.7	2.1	1.0	0.860		0.7	0.045	*
A_0046	GDP	3.9	0.7	4.0	0.8	2.8	0.7	1.0	0.802		1.4	0.023	*
A_0047	Lactic acid	8,907	2,165	7,436	1,410	10,282	2,374	1.2	0.198		0.7	0.035	*
A_0048	ADP	187	106	144	56	124	63	1.3	0.400		1.2	0.572	
A_0049	GTP	128	6.9	151	3.5	111	6.1	0.8	1.0E-04	***	1.4	6.2E-07	***
A_0050	Glyoxylate	N.A.	N.A.	N.A.	N.A.	N.A.	N.A.	N.A.	N.A.		N.A.	N.A.	
A_0051	ATP	7,934	249	7,167	188	8,122	216	1.1	1.7E-04	***	0.9	1.1E-05	***
A_0052	Glycerol 3-phosphate	319	108	336	71	391	98	0.9	0.746		0.9	0.293	
A_0053	Glycolic acid	N.A.	N.A.	N.A.	N.A.	47	8.2	N.A.	N.A.		<1	N.A.	
A_0054	Pyruvic acid	55	18	45	17	63	28	1.2	0.354		0.7	0.208	
A_0055	*N*-Acetylglutamic acid	2.6	0.04	2.8	0.2	2.7	0.3	1.0	0.196		1.0	0.499	
A_0056	2-Hydroxyglutaric acid	3.6	1.0	5.3	1.2	20	2.0	0.7	0.026	*	0.3	3.3E-07	***
A_0057	Carbamoylphosphate	N.A.	N.A.	N.A.	N.A.	N.A.	N.A.	N.A.	N.A.		N.A.	N.A.	
A_0058	Succinic acid	157	49	232	82	142	42	0.7	0.093		1.6	0.046	*
A_0059	Malic acid	168	59	241	99	175	51	0.7	0.161		1.4	0.188	
A_0060	2-Oxoglutaric acid	N.A.	N.A.	N.A.	N.A.	N.A.	N.A.	N.A.	N.A.		N.A.	N.A.	

ID consists of analysis mode and number. 'C' and 'A' showed cation and anion modes, respectively.
N.D. (Not Detected): The target peak or metabolite was below detection limits.
N.A. (Not Available): The calculation was impossible because of insufficience of the data.
¶ The ratio is of computed by using averaged detection values. The latter was used as denominator.
‖ The p-value is computed by Welch's t-test. (*<0.05, **<0.01, ***<0.001)
The data are sorted by ID in ascending order.

Supplemental Table 1: Absolute Concentration of 116 Compounds

ID	HMT DB	Concentration (nmol/g)						Comparative Analysis					
	Compound name	KO-Treatment		KO		WT		KO-Treatment vs KO			KO vs WT		
		Mean	S.D.	Mean	S.D.	Mean	S.D.	Ratio ¶	*p*-value ‖		Ratio ¶	*p*-value ‖	
A_0061	Fumaric acid	62	17	106	31	59	15	0.6	0.018	*	1.8	0.013	*
A_0062	Citric acid	159	21	233	26	161	18	0.7	3.4E-04	***	1.4	3.8E-04	***
A_0063	*cis*-Aconitic acid	1.5	0.4	2.2	0.5	1.5	0.2	0.7	0.020	*	1.5	0.016	*
A_0064	Isocitric acid	N.A.	N.A.	N.A.	N.A.	N.A.	N.A.	N.A.	N.A.		N.A.	N.A.	
C_0001	Urea	5,551	1,006	4,799	857	6,610	1,046	1.2	0.194		0.7	0.009	**
C_0002	Gly	1,601	98	1,502	323	2,142	249	1.1	0.501		0.7	0.004	**
C_0003	Putrescine	0.2	0.3	1.0	0.4	0.9	0.4	0.2	0.080		1.1	0.686	
C_0004	β-Ala	13	1.4	14	1.2	27	2.6	1.0	0.490		0.5	1.1E-05	***
C_0005	Sarcosine	12	3.3	13	3.5	13	2.0	0.9	0.836		1.0	0.978	
C_0006	Ala	2,150	323	2,119	349	1,397	256	1.0	0.875		1.5	0.003	**
C_0007	γ-Aminobutyric acid	N.A.	N.A.	N.A.	N.A.	N.A.	N.A.	N.A.	N.A.		N.A.	N.A.	
C_0008	*N*,*N*-Dimethylglycine	4.8	1.0	5.4	1.4	4.6	0.6	0.9	0.536		1.2	0.409	
C_0009	Choline	41	23	23	17	N.A.	N.A.	1.8	0.214		1<	N.A.	
C_0010	Ser	275	25	252	51	224	28	1.1	0.353		1.1	0.277	
C_0011	Carnosine	616	62	529	14	1,859	90	1.2	0.017	*	0.3	1.9E-07	***
C_0012	Creatinine	59	9.6	45	4.2	58	2.8	1.3	0.012	*	0.8	1.6E-04	***
C_0013	Pro	86	29	57	24	59	14	1.5	0.103		1.0	0.898	
C_0014	Betaine	34	6.8	31	25	11	7.9	1.1	0.744		2.9	0.109	
C_0015	Val	205	49	167	35	186	28	1.2	0.153		0.9	0.300	
C_0016	Thr	219	37	208	37	192	27	1.1	0.590		1.1	0.440	
C_0017	Homoserine	N.A.	N.A.	N.A.	N.A.	N.A.	N.A.	N.A.	N.A.		N.A.	N.A.	
C_0018	Betaine aldehyde	N.A.	N.A.	N.A.	N.A.	N.A.	N.A.	N.A.	N.A.		N.A.	N.A.	
C_0019	Cys	N.A.	N.A.	N.A.	N.A.	N.A.	N.A.	N.A.	N.A.		N.A.	N.A.	
C_0020	Hydroxyproline	88	23	35	15	165	23	2.5	0.001	**	0.2	1.8E-06	***
C_0021	Creatine	16,237	1,323	16,165	1,010	16,769	2,025	1.0	0.919		1.0	0.534	
C_0022	Leu	149	41	136	33	105	15	1.1	0.565		1.3	0.079	
C_0023	Ile	95	23	78	19	73	11	1.2	0.207		1.1	0.583	
C_0024	Asn	65	18	76	22	45	5.6	0.9	0.373		1.7	0.018	*
C_0025	Ornithine	37	5.2	31	6.2	26	2.9	1.2	0.085		1.2	0.106	
C_0026	Asp	368	61	437	135	223	34	0.8	0.288		2.0	0.010	*
C_0027	Homocysteine	N.A.	N.A.	N.A.	N.A.	N.A.	N.A.	N.A.	N.A.		N.A.	N.A.	
C_0028	Adenine	3.1	0.5	3.4	0.8	3.0	0.3	0.9	0.551		1.1	0.331	
C_0029	Hypoxanthine	N.A.	N.A.	9.9	N.A.	N.A.	N.A.	<1	N.A.		1<	N.A.	
C_0030	Spermidine	1.1	0.3	2.2	0.8	2.6	0.7	0.5	0.024	*	0.8	0.356	
C_0031	Gln	2,036	95	2,289	260	1,742	232	0.9	0.064		1.3	0.003	**
C_0032	Lys	1,458	163	1,601	537	596	172	0.9	0.555		2.7	0.005	**
C_0033	Glu	767	241	1,041	290	726	182	0.7	0.106		1.4	0.053	
C_0034	Met	57	4.4	52	9.6	56	7.7	1.1	0.314		0.9	0.445	
C_0035	Guanine	N.A.	N.A.	N.A.	N.A.	N.A.	N.A.	N.A.	N.A.		N.A.	N.A.	
C_0036	His	189	22	173	22	140	13	1.1	0.227		1.2	0.011	*
C_0037	Carnitine	415	28	419	49	292	29	1.0	0.858		1.4	5.7E-04	***
C_0038	Phe	84	17	76	9.9	73	9.4	1.1	0.317		1.0	0.648	
C_0039	Arg	379	56	456	70	185	55	0.8	0.063		2.5	3.0E-05	***
C_0040	Citrulline	113	6.5	114	13	93	11	1.0	0.876		1.2	0.011	*
C_0041	Tyr	69	11	65	6.7	83	11	1.1	0.445		0.8	0.006	**
C_0042	*S*-Adenosylhomocysteine	4.2	0.3	4.2	0.4	4.6	0.2	1.0	0.882		0.9	0.207	
C_0043	Spermine	2.7	0.3	2.7	0.12	2.3	0.2	1.0	0.958		1.2	0.147	
C_0044	Trp	25	3.3	28	4.6	29	2.8	0.9	0.239		1.0	0.707	
C_0045	Cystathionine	N.A.	N.A.	N.A.	N.A.	N.A.	N.A.	N.A.	N.A.		N.A.	N.A.	
C_0046	Adenosine	9.3	3.7	11	4.2	6.1	1.8	0.8	0.434		1.8	0.031	*
C_0047	Inosine	14	2.9	27	7.5	13	2.5	0.5	0.006	**	2.1	0.004	**
C_0048	Guanosine	2.0	0.2	2.4	0.2	N.A.	N.A.	0.8	0.017	*	1<	N.A.	
C_0049	Argininosuccinic acid	5.1	0.6	6.7	1.2	3.8	0.2	0.8	0.020	*	1.8	0.002	**
C_0050	Glutathione (GSSG)	335	52	399	88	234	81	0.8	0.164		1.7	0.007	**
C_0051	Glutathione (GSH)	299	96	300	111	376	146	1.0	0.992		0.8	0.332	
C_0052	*S*-Adenosylmethionine	22	0.7	26	2.2	20	0.7	0.9	0.007	**	1.3	7.5E-04	***

ID consists of analysis mode and number. 'C' and 'A' showed cation and anion modes, respectively.
N.D. (Not Detected): The target peak or metabolite was below detection limits.
N.A. (Not Available): The calculation was impossible because of insufficience of the data.
¶ The ratio is of computed by using averaged detection values. The latter was used as denominator.
‖ The p-value is computed by Welch's t-test. (*<0.05, **<0.01, ***<0.001)
The data are sorted by ID in ascending order.
