# Supplementary material for: Enzyme Replacement Therapy Can Reverse Pathogenic Cascade in Pompe Disease
Source: Mol Ther Methods Clin Dev. 2020 Jun 10;18:199–214. doi: 10.1016/j.omtm.2020.05.026 (PMC7334420; doi:10.1016/j.omtm.2020.05.026)
Supplement: Document S2. Article plus Supplemental Information [file mmc3.pdf]

Molecular Therapy
Methods & Clinical Development
Original Article

Enzyme Replacement Therapy Can Reverse Pathogenic Cascade in Pompe Disease

Naresh Kumar Meena,[1] Evelyn Ralston,[2] Nina Raben,[1] and Rosa Puertollano[1]

[1]Cell and Developmental Biology Center, National Heart, Lung, and Blood Institute, NIH, Bethesda, MD, USA; [2]Light Imaging Section, National Institute of Arthritis and Musculoskeletal and Skin Diseases, NIH, Bethesda, MD, USA

Pompe disease, a deficiency of glycogen-degrading lysosomal acid alpha-glucosidase (GAA), is a disabling multisystemic illness that invariably affects skeletal muscle in all patients. The patients still carry a heavy burden of the disease, despite the currently available enzyme replacement therapy. We have previously shown that progressive entrapment of glycogen in the lysosome in muscle sets in motion a whole series of "extra-lysosomal" events including defective autophagy and disruption of a variety of signaling pathways. Here, we report that metabolic abnormalities and energy deficit also contribute to the complexity of the pathogenic cascade. A decrease in the metabolites of the glycolytic pathway and a shift to lipids as the energy source are observed in the diseased muscle. We now demonstrate in a pre-clinical study that a recently developed replacement enzyme (recombinant human GAA; AT-GAA; Amicus Therapeutics) with much improved lysosome-targeting properties reversed or significantly improved all aspects of the disease pathogenesis, an outcome not observed with the current standard of care. The therapy was initiated in GAA-deficient mice with fully developed muscle pathology but without obvious clinical symptoms; this point deserves consideration.

INTRODUCTION

Following the success of enzyme replacement therapy (ERT) for the treatment of Gaucher disease in 1991,[1] this type of treatment has been developed for other lysosomal storage disorders (LSDs). ERT is now commercially available for a number of LSDs, including Pompe disease (also known as "type II glycogen storage disease"), a severe neuromuscular disorder caused by a partial or total loss of activity of the lysosomal enzyme acid alpha-glucosidase (GAA), which is solely responsible for the hydrolysis of glycogen to glucose in the lysosome. The failure to metabolize intralysosomal glycogen leads to pathological accumulation of glycogen in multiple tissues, but cardiac and skeletal muscles are most severely affected. Both hypertrophic cardiomyopathy and skeletal muscle myopathy are observed in patients with complete or near complete enzyme deficiency; this most severe infantile onset form, if left untreated, is deadly within the first year of life due to cardiorespiratory failure. Skeletal muscle myopathy eventually leading to respiratory insufficiency is the predominant manifestation of partial enzyme deficiency in patients with less severe late-onset disease.[2,3]

The introduction of ERT (alglucosidase alfa; Myozyme/Lumizyme, Sanofi Genzyme, Cambridge, MA, USA) over a decade ago has changed the natural course of the disease, primarily because of its notable effect on cardiac muscle: the infantile-onset patients live significantly longer but develop new myopathic and neurological abnormalities.[4,5] The benefit of the therapy in late-onset patients can be summarized as modest improvement/stabilization of the disease progression in the first couple of years after the ERT initiation followed by a slow decline in muscle function and respiratory insufficiency.[6–8] Several factors account for skeletal muscle resistance to the currently available ERT, including immune response (particularly in those with no residual enzyme activity) and, perhaps most importantly, inefficient muscle targeting of the recombinant enzyme (reviewed in Kohler et al.[9]). A newly developed recombinant human GAA (rhGAA) with much improved muscle targeting properties (Amicus proprietary rhGAA) appears to overcome at least some of the shortcomings of the current treatment with alglucosidase alfa. Our preclinical experiments demonstrated that this rhGAA, co-administered with miglustat (a combination that is referred to as AT-GAA) was superior to alglucosidase alfa in every measured outcome—muscle GAA uptake and activity, glycogen clearance, lysosomal size, and muscle strength.[10] Moreover, unlike alglucosidase alfa, AT-GAA alleviated autophagic defect and markedly reduced the number of fibers with autophagic buildup—a prominent feature of the diseased muscle.[9,11] We have previously shown that in addition to the defective autophagy, the pathogenic cascade leading to inexorable skeletal muscle damage and atrophy in Pompe disease involves the imbalance between protein synthesis and degradation, and dysregulation of two major signaling pathways that are linked to the lysosome and regulated by nutrient-sensing kinases—AMP-activated protein kinase (AMPK) and mammalian target of rapamycin (mTORC1).[12,13]

Here, we have extended our previous study[10] to systematically analyze the potential of AT-GAA to reverse not only the primary

Received 16 April 2020; accepted 27 May 2020;
https://doi.org/10.1016/j.omtm.2020.05.026.

Correspondence: Nina Raben, Cell and Developmental Biology Center, National Heart, Lung, and Blood Institute, NIH, Bethesda, MD, USA.
E-mail: rabenn@mail.nih.gov
Correspondence: Rosa Puertollano, Cell and Developmental Biology Center, National Heart, Lung, and Blood Institute, NIH, Bethesda, MD, USA.
E-mail: puertolr@nhlbi.nih.gov

This is an open access article under the CC BY license (http://creativecommons.org/licenses/by/4.0/).

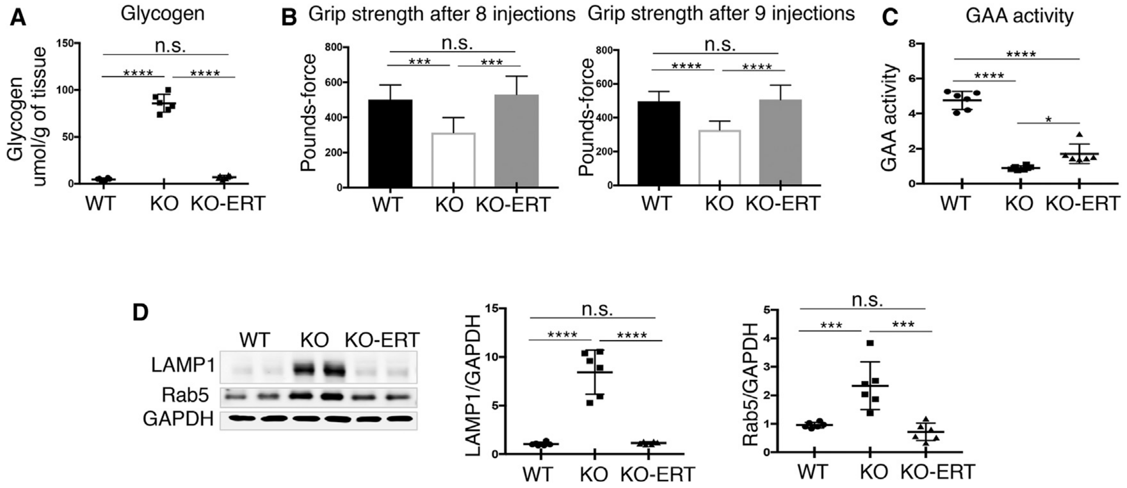

Figure 1. AT-GAA Cleared Glycogen Accumulation and Restored Normal Levels of Endosomal/Lysosomal Membrane Markers in Muscle from KO Mice

Muscle biopsies were collected from age and sex-matched WT, untreated (KO), and AT-GAA-treated KO (KO-ERT) mice. (A) Glycogen content in muscles from WT, untreated KO (KO), and treated KO (KO-ERT) mice (n = 6 for each group). (B) Muscle strength was assessed using grip-strength test after 8 and 9 administrations of the compound (n = 5 WT; n = 4 KO; n = 7 KO-ERT). (C) GAA activity in muscle tissues. (D) Western blot analysis of muscle lysates from WT, untreated KO (KO), and treated KO (KO-ERT) mice (n = 6 for each group) with the indicated antibodies. GAPDH was used as loading control. Each data point represents an individual mouse. Statistical significance was determined by one-way ANOVA. Graphs represent mean ± SD. *p < 0.05; **p < 0.01; ***p < 0.001; ****p < 0.0001.

defect—lysosomal glycogen accumulation—but also a chain of secondary downstream events stemming from the lysosomal dysfunction in muscle of GAA knockout (KO) mice. We have looked at the long-term effect of AT-GAA on a range of lysosomal/autophagic markers, mTORC1-dependent and independent protein synthesis and proteasomal degradation, as well as on the upstream regulators and downstream targets of AMPK and mTORC1. Furthermore, we found clear metabolic differences between the wild-type (WT) and KO animals, suggesting a decreased glucose availability in the glycolytic GAA-deficient muscle. This unexpected result sheds further light on the metabolic consequences of the lysosomal glycogen accumulation in the diseased muscle. Our data indicate that the therapeutic benefit of AT-GAA is unequivocal; the therapy significantly improved or reversed multiple aspects of the disease pathogenesis, thus offering clear advantage over the available therapy.

RESULTS

Effect of AT-GAA on Lysosomal and Autophagic Pathologies

A combination of rhGAA (ATB200; Amicus proprietary; 20 mg/kg) with the small-molecule pharmacological chaperone (AT2221; miglustat; 10 mg/kg) was used to treat GAA-KO mice (KO). The benefits of coadministration of ATB200/AT2221 (referred to as AT-GAA) were described previously.[10] KO mice received 11–12 biweekly intravenous (i.v.) administrations of AT-GAA; the animals were 3–4 months old at the start of therapy, and they were sacrificed 7–10 days after the last administration at the age of 8–9 months. The therapy completely reversed excess glycogen accumulation and restored muscle strength, as indicated by the measurements of the maximum force displayed by the animals during the grip strength test (Figures 1A and 1B). The levels of GAA activity in muscle of treated animals were above the background levels seen in the KO mice; these levels, however, remained lower than in the WT controls, which is not surprising considering the timing for collecting the samples (Figure 1C). The levels of both endosomal (Rab5) and lysosomal (LAMP1) markers that are significantly increased in the affected muscle returned to the WT levels in treated KO animals (Figure 1D). Thus, it appears that the efficient glycogen degradation following the therapy eliminates the need for more lysosomal membranes that are required to house the accumulated substrate.

We then looked at the levels of multiple proteins involved in different stages of autophagic process. Autophagic defect, namely, an increase in autophagosome number combined with the inefficient fusion between autophagosomes and lysosomes, manifests as massive buildup of cellular debris in the diseased muscle.[9] The levels of the Vps15 protein kinase, the lipid kinase catalytic subunit Vps34, and the regulatory protein Beclin1, all part of the class III phosphatidylinositol 3-kinase complex I, that is required for the initial steps of autophagy—autophagosome nucleation and maturation[14,15]—were increased in KO muscle, and all three proteins were back to the WT levels following the therapy (Figure 2A). Of note, an increase in the levels of Vps15, Vps34, and Beclin1 was also found in muscle biopsies from Pompe disease patients.[16] Three other proteins, which are also directly involved in the autophagic process, were analyzed: Atg7, a protein essential for autophagosome biogenesis,[17,18] the autophagy substrate p62/SQSTM1, a marker of autophagic flux,[19] and the lipidated form of LC3 (LC3-II), a well-accepted marker of autophagosomes.[20] The treatment markedly improved the status of all three of them, although the level of Atg7 still remained increased following the treatment when compared to the WT (Figure 2A). A decrease in the autophagy markers correlated with the reduction in the amounts of accumulated high molecular weight

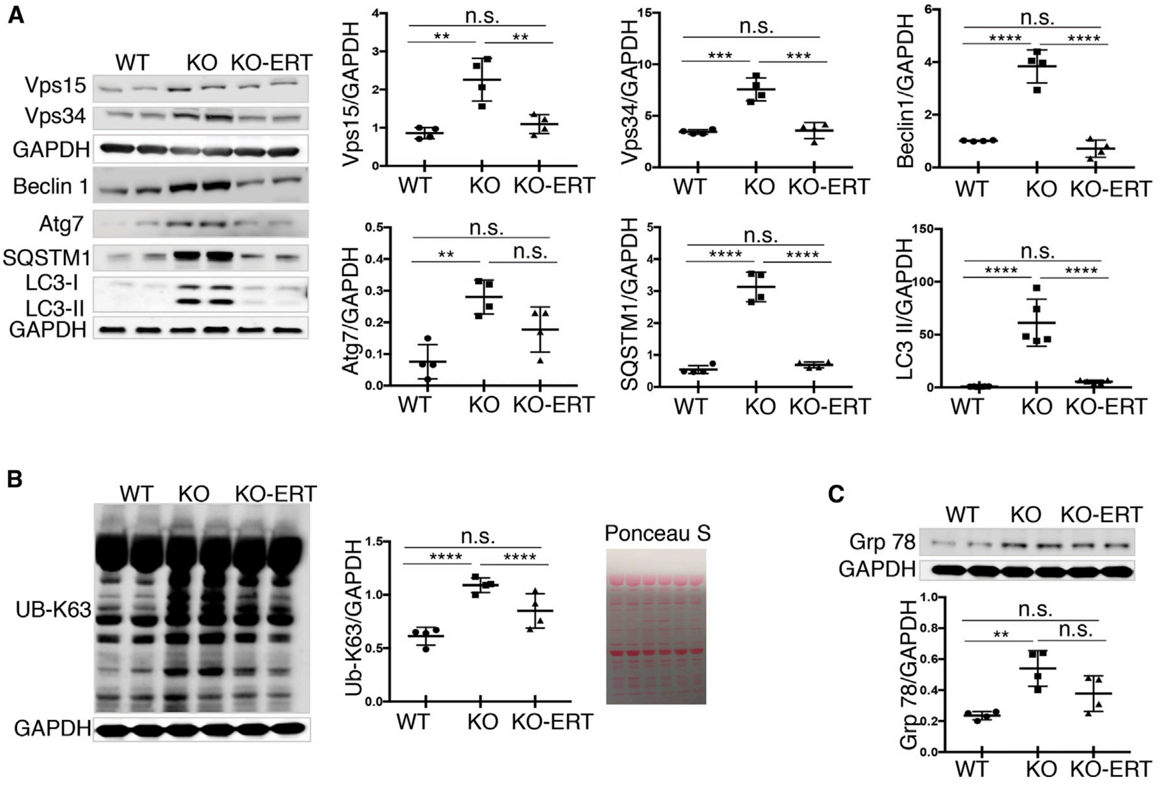

Figure 2. AT-GAA Reversed the Levels of the Autophagic Markers and Alleviated the Burden of Accumulated Protein Aggregates and ER-Stress in Muscle from KO Mice

Muscle biopsies were collected from age and sex-matched WT, untreated (KO), and AT-GAA-treated KO (KO-ERT) mice. (A) Western blot analysis of muscle lysates from WT, untreated KO (KO), and treated KO (KO-ERT) mice with the indicated antibodies (n = 4 for each group; n = 5 for western blot with LC3 antibody). (B) Western blot analysis of muscle lysates from WT, untreated KO (KO), and treated KO (KO-ERT) mice with K63-linked ubiquitin specific antibody (n = 4 for each group). Western blot with anti-GAPDH and Ponceau S staining were used as loading controls. (C) Western blot analysis of muscle lysates from WT, untreated KO (KO), and treated KO (KO-ERT) mice with anti-Grp 78 antibody (ER-stress marker; n = 4 for each group); GAPDH was used as loading control. Each data point represents an individual mouse. Statistical significance was determined by one-way ANOVA. Graphs represent mean ± SD. *$p < 0.05$; **$p < 0.01$; ***$p < 0.001$; ****$p < 0.0001$.

K63-linked Ub proteins (that are targeted for autophagy-lysosomal degradation pathway) in treated- compared to untreated KO, although their levels did not reach the control WT values (Figure 2B). In addition, glucose-regulated ER stress protein HSPA5/Grp 78 showed a tendency to decrease in treated animals, but the differences did not reach significance (Figure 2C).

Consistent with these data, immunostaining of single muscle fibers with lysosomal (LAMP1) and autophagosomal (LC3) markers demonstrated that the number of myofibers with typical buildup fell dramatically from ~100% in untreated 8- to 9-month-old KO (Figures 3B and 3C; Video S1) to less than 20% in treated KO (Figures 3D–3G). Immunostaining of muscle fibers from the treated KO mice (n = 217 fibers from four animals) revealed three distinctive patterns based on the area occupied by the buildup: (1) buildup-free normal-looking fibers (n = 115; 53%; Figure 3D); (2) near normal fibers with the remnants of the buildup area not exceeding 2–2.5 μm in diameter (n = 67; 30.9%; Figures 3E and 3F); and (3) fibers with more typical buildup, although shorter in length compared to that in the untreated KO (n = 35; 16%; Figure 3G). Only occasional fibers (less than 3%–4%) contained a typical buildup (Figure S1). These data were bolstered by examination of unstained muscle biopsies by second harmonic generation (SHG) imaging. Unlike the imaging of individual immunostained muscle fibers, SHG microscopy combined with two-photon-excited fluorescence allows visualization of myosin bands and autofluorescent lysosomes; it provides detailed structural information in images of relatively large bundles of unstained minimally processed muscle fibers.[21,22] We previously reported a range of muscle defects in virtually every fiber from KO mice.[23] A much-improved muscle architecture following the therapy is shown using this imaging (Figure 4).

These data indicate that the resolution of autophagic buildup follows lysosomal glycogen clearance but, perhaps not surprisingly, is still lagging behind. The reversal of autophagic pathology is likely to require sufficient number of "healthy" functional lysosomes able to fuse with autophagosomes and digest their content. To see whether the therapy reverses lysosomal damage, we analyzed the levels of several cytosolic galectins (galectin 1, 9, and 3) that have been shown to function as

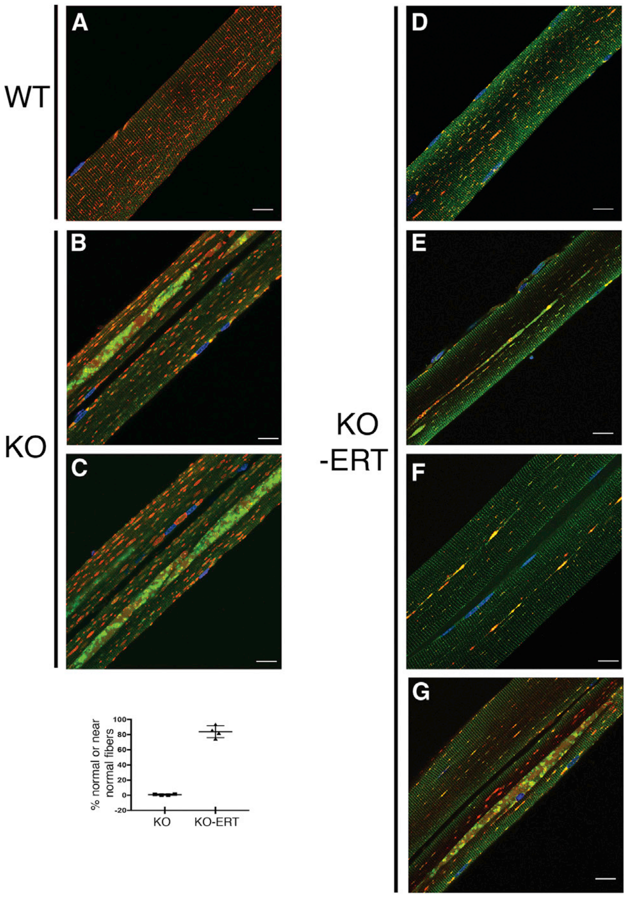

Figure 3. AT-GAA Eliminated Autophagic Buildup in Vast Majority of Myofibers from KO Mice

Muscle biopsies were collected from age and sex-matched WT, untreated (KO), and AT-GAA-treated KO (KO-ERT) mice. Confocal microscopy images of single muscle fibers immunostained with lysosomal marker LAMP1 (red) and autophagosomal marker LC3 (green); nuclei are stained with Hoechst dye (blue). (A) Abundant dot-like LAMP1-positive structures (red) are seen in a fiber from a WT mouse; LC3-positive autophagosomes are barely detectable in control fibers (n = 60 from 3 mice). (B and C) Enlarged lysosomes (red) and autophagic buildup (the multicolored areas in the core of muscle fibers) are detected in virtually all myofibers from KO mice; the images show two neighboring fibers with autophagic buildup located in different focal planes; therefore, the buildup is seen in the left fiber in (B), and in the right fiber in (C) (n = 85 from 4 untreated KO mice). See also Video S1. (D–G) The 4 right panels show the representative images of myofibers from treated KO mice (n = 217 from 4 mice). Myofibers shown in (D), as well as the left fiber in (G), are considered normal; near normal fibers are shown in (E) and (F) (n = 182; ~84% of normal or near normal fibers). The right fiber in (G) contains a more typical buildup (n = 35; ~16%). The dot plot indicates the percentage of normal/near normal fibers from treated KO compared to untreated KO mice. Graphs represent mean ± SD. Scale bars, 20 μm. (See also Figure S1).

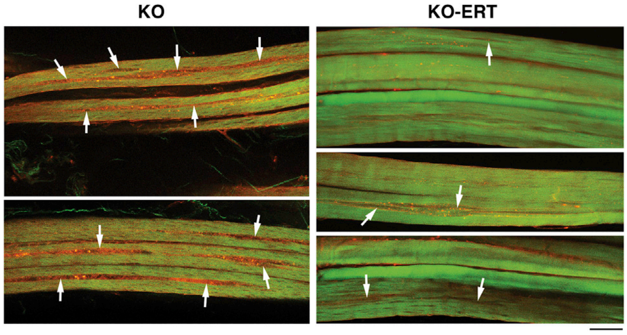

Figure 4. AT-GAA Improved Muscle Quality and Architecture in KO Mice

Muscle biopsies were collected from age and sex-matched WT, untreated (KO), and AT-GAA-treated KO (KO-ERT) mice. The samples were analyzed by second harmonic generation (SHG) and two-photon-excited fluorescence (2PEF) imaging. SHG reveals the position and organization of myosin heavy chain, while (2PEF) reveals mitochondria and autofluorescent particles. Every fiber from untreated KO mice (n = 3) show long longitudinal interruptions of the SHG image filled with 2PEF-positive particles (arrows); these areas correspond to the space occupied by the autophagic buildup. In contrast, most fibers from the treated KO mice (n = 3) do not show this defect; nevertheless, small interruptions of the SHG images can be seen in some fibers (arrows). Note that most fibers from treated mice have a larger diameter compared to that in untreated KO. Scale bars, 25 μm.

sensors of endosomal/lysosomal damage.[24,25] Immunoblotting analysis showed a significantly increased level of galectin 3 in the affected muscle (Figure 5A). Elevated level of galectin 3 in the KO muscle was detected as early as in 6-week-old mice (data not shown). Interestingly, the increase in galectin 3, a particularly sensitive marker of lysosomal damage, was observed in KO skeletal muscle but not in the diaphragm or heart, both of which are free from the autophagic buildup[26] (Figure 5B). Furthermore, no increase in galectin 3 was seen in muscle-specific autophagy-deficient KO mice; in these KO mice, referred to as double KO (DKO), autophagic buildup is abolished by the genetic inactivation of *Atg7* gene in skeletal muscle[27] (Figure 5C). Thus, galectin 3 can serve as a prognostic autophagy-related biomarker. We have also measured galectin 3 in blood of the KO mice but found a decrease rather than an increase in its level (Figure 5D); this decrease is likely a reflection of a reduced amount of galectin 3 in non-skeletal muscle tissues. Importantly, the increase of galectin 3 in KO muscle was reversed following the treatment (Figure 5A).

Of note, we previously reported an improvement in the levels of LAMP1, p62/SQSTM1, and LC3 following a short-course regimen of 4 biweekly administrations of AT-GAA in KO mice; the dosage of the drug and the animals age at the start of therapy were the same as in the current study.[10] However, all three markers were still significantly above normal in treated KO (although much lower than in untreated KO), whereas the levels of all three returned to the WT control values with longer treatment as indicated above. Thus, the drug appears to keep pace with the ongoing disease progression and continues to ease the burden of autophagic buildup; the number of fibers with autophagic defect dropped from 60% after the short-course treatment[10] to ~20% in the current study.

Effect of AT-GAA on Signaling Pathways and Protein Homeostasis

AMPK Signaling

We have previously demonstrated that the lysosomal dysfunction in GAA-deficient muscle cells leads to aberrant signaling of AMPK and

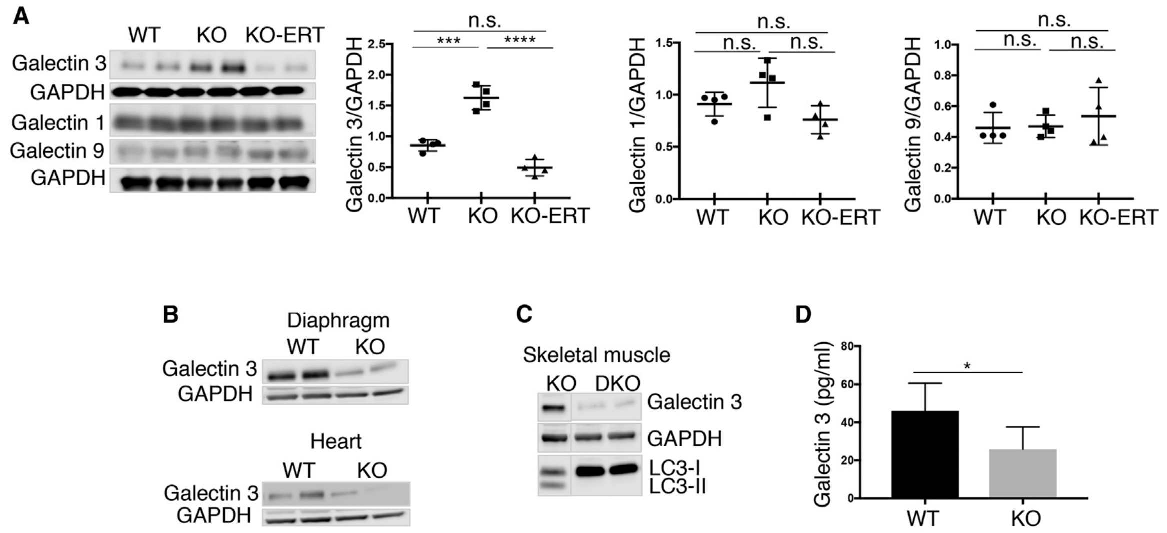

Figure 5. AT-GAA Reversed the Level of Galectin 3, a Marker of Lysosomal Damage, in Muscle from KO Mice

Muscle biopsies were collected from age and sex-matched WT, untreated (KO), and AT-GAA-treated KO (KO-ERT) mice. (A) Western blot of muscle lysates from WT, untreated KO (KO), and treated KO (KO-ERT) mice with the indicated antibodies (n = 4 for each group). Only galectin 3 was increased in muscle from untreated KO mice; the level of galectin 3 was reduced on therapy and reached the WT control value. Statistical significance was determined by one-way ANOVA. Graphs represent mean ± SD. ***$p < 0.001$; ****$p < 0.0001$. (B) Western blot of lysates from the diaphragm (top) and heart (bottom) of WT and untreated KO (KO) mice with anti-galectin 3 antibody. (C) Western blot of muscle lysates from untreated KO and muscle-specific autophagy-deficient KO mice (DKO) with anti-galectin 3 antibody. Efficient suppression of autophagy in skeletal muscle of DKO mice is indicated by the absence of LC3-II band. The blots are composite images; the samples were run on the same gel. Source data are available online for this figure. GAPDH was used as loading control. (D) Quantification of galectin 3 in serum from the WT and KO mice by ELISA. Student's t test was used for statistical analysis. Data are mean ± SD. *$p < 0.05$ (n = 6).

mTORC1,[12,13] the two major nutrient-sensing kinases, which cooperate and exert opposite regulatory effects on cellular metabolism. Consistent with our previous data,[12] activation of AMPK and a rise in the level of its upstream activation kinase, liver kinase B1 (LKB1) were observed in muscle from KO mice (Figure 6A). Following the treatment, both the LKB1 level and AMPK activity, measured by the LKB1-mediated Thr[172] phosphorylation in its catalytic domain, decreased significantly and reached the WT levels (Figure 6A).

We then looked at the phosphorylation levels of a number of AMPK downstream targets to see whether the treatment restores the LKB1-AMPK pathway. In skeletal muscle, this pathway regulates both glucose and lipid metabolism and, once activated, results in increase in fatty acid oxidation and glucose transport. Lipid metabolism is controlled by inhibitory phosphorylation of acetyl coenzyme A (CoA) carboxylase (ACC Ser[79]) leading to the increased uptake of fatty-acyl-CoA into the mitochondria for oxidation. Glucose uptake into skeletal muscle is controlled by AMPK-mediated translocation of the glucose transporter 4 (GLUT4) from intracellular sites to plasma membrane. This process was shown to involve TBC1D1, a Rab-GTPase-activating protein (Rab-GAP) that is highly expressed in glycolytic muscle; AMPK-directed inhibitory phosphorylation of a key regulatory site, TBC1D1 Ser[660], is associated with the increase in muscle glucose uptake[28,29] (reviewed in Cartee et al.[30]).

Indeed, a significant increase in phosphorylation of both ACC and TBC1D1 (inactivation) was observed in KO muscle. Correspondingly, the levels of GLUT4 were markedly increased in the affected muscle (Figure 6B); this finding is consistent with our previous data showing an insulin-independent increase in the GLUT4 level at the plasma membrane and elevated basal glucose uptake in skeletal muscles of KO mice.[31] The phosphorylation levels of ACC and TBC1D1, as well as the amount of GLUT4, returned to normal following the treatment (Figure 6B).

Autophagy is yet another process that is under the control of AMPK. When active, AMPK stimulates autophagy directly by phosphorylating autophagy-initiation kinase ULK-1 and indirectly by negative regulation of mTORC1 (a major inhibitor of autophagy) through phosphorylation and activation of TSC2.[32–35] Consistent with our previous reports,[12,13] AMPK-mediated phosphorylation of TSC2^{S1387} and ULK-1^{S317} were markedly increased in KO muscle (Figure 6C). Following the treatment, the amount of phosphorylated ULK-1^{S317} returned to normal, whereas the levels of TSC2^{S1387} showed only a modest tendency to decrease and remained elevated compared to the WT (Figure 6C). A diagram in Figure 6D shows the upstream input and downstream AMPK targets, which were analyzed in this study.

Taken together, these data indicate that activation of AMPK, a key cellular sensor of energy stress and glucose levels (reviewed in Carroll and Dunlop[36]), in the KO muscle turns on several programs to supplement ATP production. However, these protective mechanisms may affect glycogen synthesis and exacerbate lysosomal glycogen storage and autophagic buildup. The treatment appears to largely

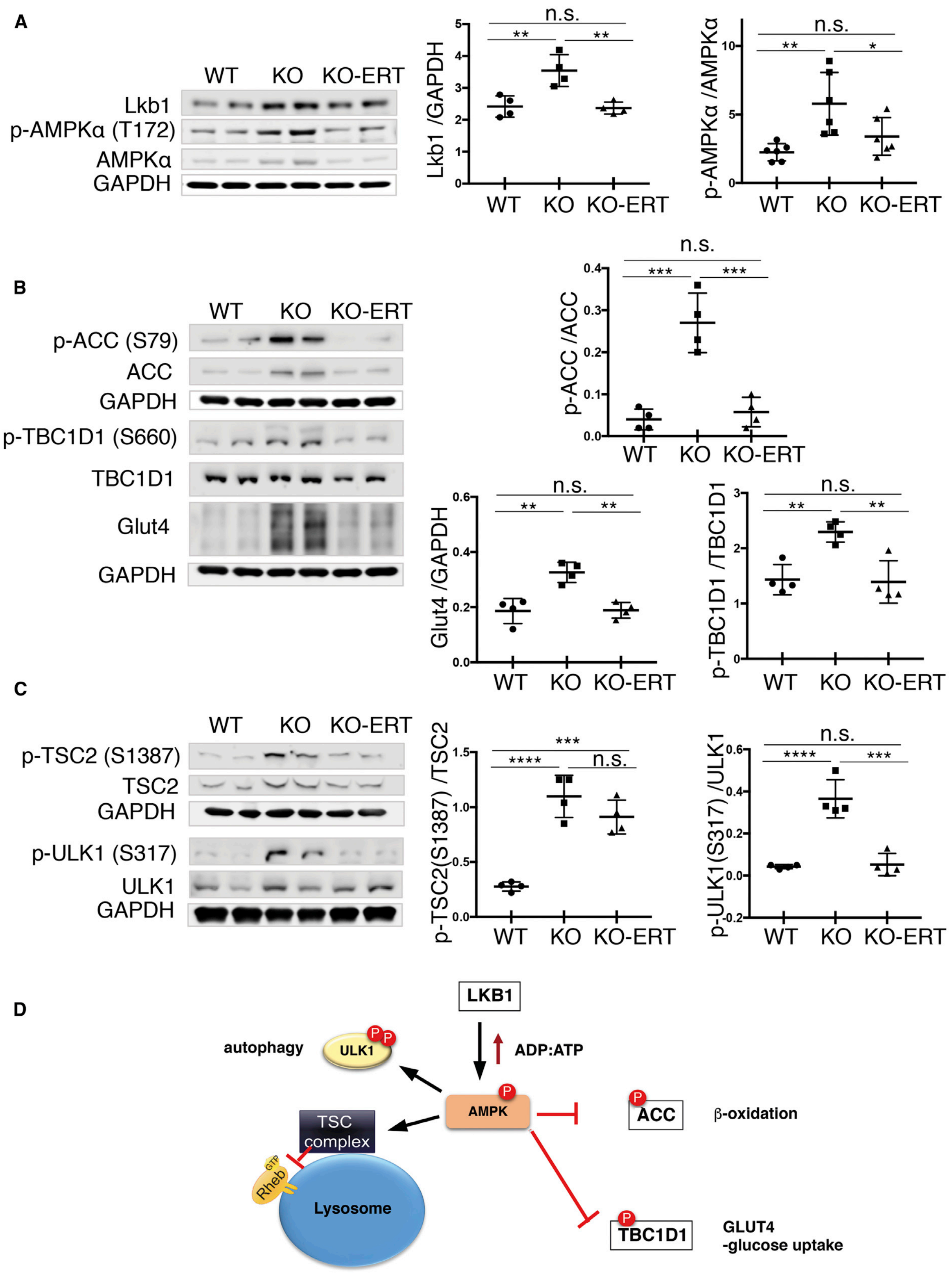

(legend on next page)

restore these signaling pathways with a notable exception of AMPK-TSC2 axis, which exerts an inhibitory effect on mTORC1 by inhibiting Rheb, a powerful activator of mTORC1 at the lysosomal surface[37–39] (reviewed in Laplante and Sabatini[40]).

mTORC1 Signaling

In agreement with our previous data,[12] no significant changes in the levels of active phosphorylated AKT^{S473} (an upstream regulator of mTORC1) were seen in KO muscle, whereas the ratio of phosphorylated PRAS40^{T246}/total (proline-rich AKT substrate of 40 kDa; a downstream target of AKT) was decreased in the KO compared to the WT (Figure 7A). Since AKT-mediated phosphorylation of PRAS40 is known to relieve the inhibitory effect of PRAS40 on mTORC1,[41] a relative increase in hypophosphorylated PRAS40 is consistent with the inhibition of mTORC1 activity in the KO, a condition which was much improved following the treatment; the p-PRAS40^{T246}/total ratio was increased and reached near-normal levels (Figure 7A).

To further investigate the effect of the therapy on the mTORC1 signaling, we looked at the phosphorylation state of mTORC1 downstream effectors, the inhibitory translation initiation factor 4EBP1, and the ribosomal protein 6 (S6), which is phosphorylated by the p70 S6 kinase, a direct mTORC1 substrate. The ratios of both p-4EBP1$^{T37/46}$/total and p-4EBP1^{S65}/total, as well as the ratio of p-S6$^{S235/236}$/total were markedly reduced in the KO muscle and remained reduced despite the treatment, suggesting a diminished mTORC1 activity in the affected muscle (Figure 7B). However, the actual levels of the phosphorylated forms and the total amount of mTORC1 were greatly reduced in muscle from treated compared to untreated KO mice; perhaps, more importantly, the levels of the non--phosphorylated active 4EBP1 form dropped significantly following the treatment. Hypophosphorylated 4EBP1 binds to eIF4E and represses cap-dependent translation by blocking the formation of the active eIF4E·eIF4G complex (reviewed in Hay and Sonenberg[42]). Thus, a decrease in the non-phosphorylated 4EBP1 in muscle from the treatment group suggests an improvement in the mTORC1 signaling. A diagram in Figure 7C shows the upstream input and downstream mTORC1 targets that were analyzed in this study. Importantly, a similar improvement was already observed after the shortened treatment regimen of 4 administrations of the drug (Figure S2), indicating that the extended therapy maintained the condition without further improvement, and that the full correction of mTORC1 signaling may require additional intervention.

Protein Homeostasis

Previously, we demonstrated that protein synthesis in KO muscle was enhanced despite a diminished mTORC1 activity; this increase was accounted for by a significant decrease in the stress- and metabolite-induced phosphorylation of the alpha subunit of the eukaryotic translation initiation factor 2 (eIF2α/EIFS1).[13] When EIF2S1 is phosphorylated at serine 51, it locks eIF2 in an inactive state, leading to the inhibition of general translation initiation (reviewed in Aitken and Lorsch[43]). These results were replicated in the current study: the rate of protein synthesis, measured *in vivo* by the nonradioactive surface sensing of translation (SUnSET) method, was increased, whereas the level of p-EIF2S1^{S51} was decreased in KO muscle (Figures 8A and 8B). The SUnSET method relies on the incorporation of puromycin, a structural tyrosyl-tRNA analog, into nascent peptide chains leading to the termination of their elongation.[44] Western blotting with an anti-puromycin antibody was performed using muscle collected 30 min after intraperitoneal injection of puromycin (0.04 μmol/g). A similar increase in anti-puromycin immunoreactivity and a decrease in p-EIFS1^{S51} were observed following the treatment (Figures 8A and 8B). This apparent absence of the therapeutic effect is not necessarily a negative sign, because the increase in muscle proteolysis in KO was not fully reversed in treated KO mice, as indicated by the levels of proteasomal subunits (PSMC1 and PSMA5) and the chymotrypsin-, trypsin-, and caspase-like proteasome activities (Figures 8C and 8D). Thus, the protein balance is tilted toward protein synthesis in muscle of treated compared to untreated KO mice.

Metabolic Dysfunction in KO Muscle and the Effect of AT-GAA

We have previously reported an energy deficit in KO muscle cells and suggested that this deficit may stem from reduced availability of glucose due to the failure to degrade lysosomal glycogen.[45] In addition, we recently published the results of metabolome analysis of muscle biopsies from two sets of WT and KO mice (3–4 months old, n = 5 WT, n = 7 KO; and 5–6 months old, n = 5 WT, n = 7 KO).[13] However, in this publication we focused on the levels of amino acids to support the data on increased proteolysis in the affected muscle. Here, we performed yet another metabolome analysis of muscle samples from 8- to 9-month-old WT, untreated, and ERT-treated KO mice (n = 6 per group; all females) using capillary electrophoresis mass spectrometry (CE-MS). Complete data on 116 annotated metabolites are presented in Table S1. Thus, we had at our disposal three sets of data on the metabolic changes in muscle from untreated KO mice ranging from 3 to 9 months of age. We first summarized these data and focused on the major energy-producing pathways—glycolysis and the mitochondrial tricarboxylic acid (TCA) cycle. The relative values of the detected metabolites involved in glycolysis and the TCA cycle in untreated KO and WT are indicated in Table 1.

Numerous metabolites of the glycolytic pathway showed a downward trend in all KO groups and several were significantly decreased,

Figure 6. AT-GAA Improved AMPK-TSC2 Signaling in Muscle from KO Mice

Muscle biopsies were collected from age and sex-matched WT, untreated (KO), and AT-GAA-treated KO (KO-ERT) mice. (A) Western blot analysis of muscle lysates from WT, untreated KO (KO), and treated KO (KO-ERT) mice with the indicated antibodies (n = 4 for each group for LKB1; n = 6 for each group for p-AMPKα T^{172}). (B and C) Western blot analysis of muscle lysates from WT, untreated KO (KO), and treated KO (KO-ERT) mice with the indicated antibodies (n = 4 for each group). GAPDH was used as loading control. Each data point represents an individual mouse. (D) A diagram showing the position of the analyzed proteins. Statistical significance was determined by one-way ANOVA. Graphs represent mean ± SD. *p < 0.05; **p < 0.01; ***p < 0.001; ****p < 0.0001.

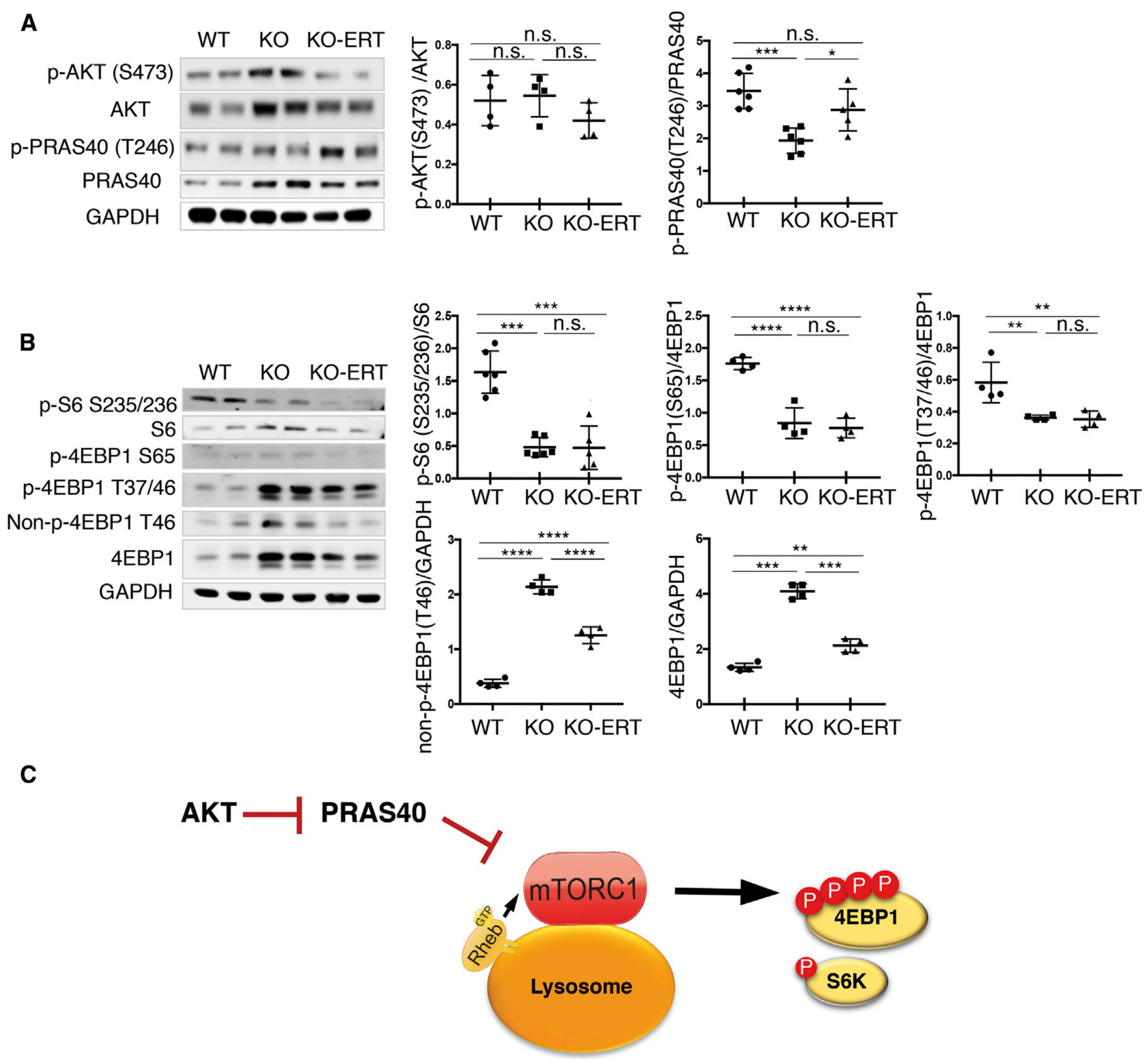

Figure 7. AT-GAA Had a Modest Effect on mTORC1 Signaling in Muscle from KO Mice

Muscle biopsies were collected from age and sex-matched WT, untreated (KO), and AT-GAA-treated KO (KO-ERT) mice. (A) Western blot analysis of muscle lysates from WT, untreated KO (KO), and treated KO (KO-ERT) mice with anti-AKT and anti-PRAS40 antibodies (n = 4–6 for each group). (B) Western blot analysis of muscle lysates from WT, untreated KO (KO), and treated KO (KO-ERT) mice with the indicated antibodies (n = 4–6 for each group). Different forms of 4EBP1, phosphorylated (p-4EBP1$^{T37/46}$ and p-4EBP1^{S65}), non-phosphorylated (non-p-4EBP1^{T46}), and total were analyzed. No changes in the mTOR activity are detected in treated compared to untreated KO samples; both exhibit diminished mTOR activity when compared to WT as shown by the decrease in the levels of phosphorylated S6 and 4EBP1, downstream targets of mTORC1 (top panels). A decrease in both non-p-4EBP1^{T46} and total is seen in treated compared to untreated KO muscle (lower panels). GAPDH was used as loading control. Each data point represents an individual mouse. (C) A diagram showing the position of the analyzed proteins. Statistical significance was determined by one-way ANOVA. Graphs represent mean ± SD. *$p < 0.05$; **$p < 0.01$; ***$p < 0.001$; ****$p < 0.0001$.

particularly in the older groups when compared to the WT. These include glucose-1-phosphate (G1P), glucose-6-phosphate (G6P), fructose-6-phosphate (F6P), fructose-1,6-bisphosphate, pyruvate, and lactate. These data indicate a decrease in glycolytic capacity in the diseased muscle. Pyruvate, the end product of glycolysis, is converted either to lactate or to acetyl-CoA for ATP production via mitochondrial oxidative phosphorylation. However, the levels of acetyl-CoA in KO muscle were significantly increased, suggesting a shift to fatty acid metabolism. The degradation of fatty acids in the mitochondria generates acetyl-CoA, which enters the TCA cycle. The increase in acetyl-CoA in KO muscle correlated with activation of the TCA cycle, as indicated by the increase in its intermediates—citrate, succinate, malic acid, fumarate, and malate. These data are supported by the increase in carnitine, which is utilized as a shuttle mechanism to transport activated fatty acids (acyl-CoAs) into the mitochondrial matrix, where β-oxidation takes place.[46] Thus, it appears that skeletal muscle in the KO switched from carbohydrates to lipids as the main energy source since glucose availability is limited. This novel finding

Table 1. Changes in the Metabolite Levels in Skeletal Muscle in KO Compared to WT Mice

Compound	KO/WT-f 3–4 Months Old	KO/WT-m 5–6 Months Old	KO/WT-f 8–9 Months Old
Glycolysis			
G1P	0.8	0.6**	0.6*
G6P	0.9	0.7***	0.8*
Fructose 6-phosphate	0.8	0.7***	0.7**
Fructose 1,6-diphosphate	0.9	0.5**	0.7
3-Phosphoglyceric acid (3PG)	0.8	0.6***	1.0
2-Phosphoglyceric acid (2PG)	0.8	0.6***	1.0
Pyruvic acid	0.8*	0.8***	0.7
Lactic acid	0.7**	0.7**	0.7*
Acetyl-CoA	1.3**	1.4*	1.7*
TCA (Krebs Cycle)			
Citric acid	1.7***	1.1*	1.4***
Cis-aconitic acid	-	1.8*	1.5*
Succinic acid	1.2	1.3	1.6*
Fumaric acid	1.5**	1.9**	1.8*
Malic acid	1.2	1.6**	1.4
Carnitine	1.9***	1.5***	1.4***
Glycogen Synthesis Precursors			
Galactose 1-phosphate	2.0**	1.7	2.8**
UDP-glucose	1.7***	1.6***	1.6*

may help in the development of tailored nutrition therapy. In addition, we have found a consistent increase in glycogen synthesis precursors in KO muscle, galactose 1-phosphate, and UDP-glucose (the immediate glucose donor for glycogen synthesis), suggesting inhibition of cytosolic glycogen synthesis (Table 1).

The changes in the overall metabolite profile of muscle from WT, untreated, and treated KO mice are shown in Figure 9. Principal-component analysis showed a clear separation between the WT and KO muscle samples; metabolites of the ERT-treated KO mice comprised a third group (Figure 9A). Similarly, the hierarchical clustering analysis (hit map analysis) of muscle samples from individual WT, untreated, and treated KO revealed a clear clustering of metabolomic changes in each of the three conditions (Figure 9B). Treatment shifted the metabolic profile of KO mice closer to that of the WT, reflecting a positive response.

Most metabolic parameters of glycolysis and the TCA cycle were improved in the treated KO muscle, and some, in particular those of the TCA cycle, reached the WT levels (Figures 10A and 10B). However, the level of carnitine remained increased in muscle from treated KO mice (Figure 10C). Consistent with our previous data, the ATP level was decreased, whereas the amino acid content was increased in untreated KO muscle; the treatment largely reversed the energy deficit and only marginally reduced the level of total amino acids. In addition, the treatment had a "normalizing" effect on glycogen synthesis, as shown by a decrease in the amount of the accumulated precursors (Figure 10D) and a decrease in the level of phosphorylated glycogen synthase (p-GS^{S641}; activation) in treated compared to untreated KO mice (Figure 10E).

In summary, we have demonstrated for the first time that the limited availability of glucose as a source of energy in KO muscle is associated with a shift in fuel selection toward lipid oxidation, and that the treatment ameliorated these metabolic abnormalities. Our data also indicate that there is a crosstalk between the cytosolic and lysosomal glycogen degradation. Finally, it is important to emphasize that the lysosomal and autophagic pathologies in KO muscle develop very early, well before the clinical symptoms; these changes were already detectable in 10-day-old KO mice (Figure S3). Therefore, an efficient AT-GAA therapy, may become curative if initiated early enough.

DISCUSSION

Since the development of enzyme replacement therapy for Pompe disease, and more recently the advances in gene therapy, a large body of research has been conducted to assess the effectiveness of different treatments in preclinical studies in the KO models of the disease. In all these studies, the outcome measures, which are commonly used to evaluate the impact of different interventions in skeletal muscle, a major affected tissue, have been limited to monitoring glycogen clearance, GAA activity, and muscle function. More recently, subsequent to our finding of the autophagy defect in the diseased muscle (reviewed in Kohler et al.[9] and Lim et al.[11]), lysosomal and autophagic markers, such as LAMP1, LC3, and p62/SQSTM1 have been added to the arsenal. Using these metrics, we previously compared side-by-side the efficacy of AT-GAA and alglucosidase alfa, the current standard of care; AT-GAA was more effective at reversing glycogen accumulation in KO muscle and, unlike alglucosidase alfa, significantly reduced the number of myofibers with autophagic buildup after four administrations of the drug.[10]

Over the course of the last few years, we have shown that in addition to lysosomal enlargement and defective autophagy, the cellular impact of lysosomal dysfunction in KO muscle involves aberrant mTORC1 and AMPK signaling.[12,13] The experiments exploring these abnormalities in the context of Pompe disease became possible because of the basic science discoveries of the role of the lysosome as a nutrient sensor and a major platform to assemble signaling complexes that regulate cell growth and metabolism (reviewed in Settembre et al.,[47] Efeyan et al.,[48] and Perera and Zoncu[49]). Both kinases are established as the key negative (mTORC1) and positive (AMPK) regulators of autophagy, and both are recognized as major regulators of skeletal muscle metabolism.[32–35]

The pathogenic changes we observed in untreated KO muscle can be briefly summarized as follows: activation of AMPK and diminished mTORC1 activity; induction of autophagy and accumulation of

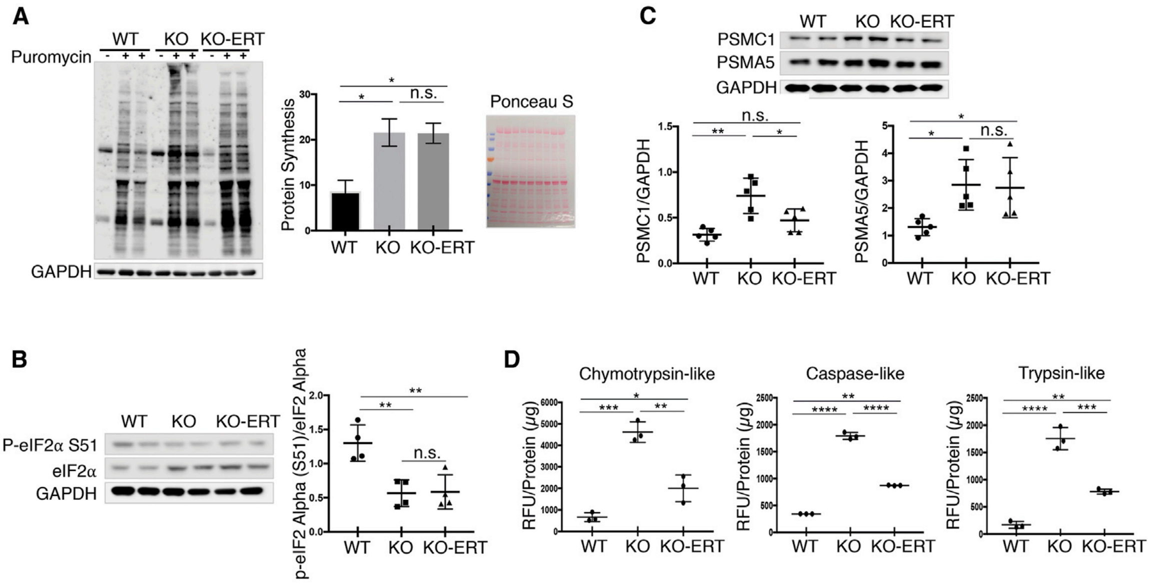

Figure 8. AT-GAA Had a Modest Effect on Muscle Proteostasis in KO Mice

KO mice received 12 bi-weekly i.v. administrations of AT-GAA. Age and sex-matched WT and untreated KO mice were used for the comparisons. Muscle biopsies were collected 7–10 days after the last administration. (A) Surface sensing of translation (SUnSET) analysis was used to evaluate the rate of protein synthesis. The animals were injected intraperitoneally (i.p.) with puromycin (the aminoacyl-tRNA analog) 30 min prior to sacrifice. Western blot of muscle lysates with anti-puromycin antibody was then used to detect the incorporation of puromycin into nascent polypeptides. Total intensity of puromycin-labeled polypeptides was quantified. The increased protein translation is observed in both untreated and treated KO samples. Western blot with anti-GAPDH and Ponceau S staining were used as loading controls. (B) Western blot of muscle lysates from WT, untreated-, and treated KO muscle shows a decrease in the p-eIF2α^{S51}/eIF2α ratio (consistent with the increase in protein synthesis) in both untreated and treated KO samples. (C) Western blot of total lysates from WT, untreated-, and treated KO muscle shows increased levels of proteasome 26S subunit, ATPase 1 (PSMC1), and alpha 5 (PSMA5) subunits in the KO; the level of PSMC1 returned to normal following ERT, whereas the level of PSMA5 did not (n = 5 for each condition. (D) The proteasome activity was measured in proteasome-enriched fractions isolated from WT, untreated-, and treated KO muscle extracts. The activity was significantly improved following ERT but still remained elevated compared to the WT controls. The results are shown in relative fluorescence units (RFU)/mg protein (n = 3 for each group). Note that the lysates for (B) and (C) were the same as those used in Figures 1, 2, 5,6, and 7 (n = 4–5 for each group). Data are mean ± SD. Statistical significance was determined by one-way ANOVA. *p < 0.05; **p < 0.01; ***p < 0.001; ****p < 0.0001.

Ub-protein aggregates; mTORC1-independent increase in protein synthesis and increased muscle proteolysis. These findings provided the framework for assessing the efficacy of AT-GAA following the long-term (5 months) treatment in KO mice. Thus, this long-term preclinical study offers the most comprehensive analysis of the effect of the drug on glycogen level, autophagy, protein synthesis and degradation, and signaling pathways. Importantly, in the course of this study, we confirmed and expanded our previous observations in untreated KO muscle.[12,13] Among the newly identified changes, the increase in galectin 3 (LGALS3/galectin 3) appears to be most informative because of its positive association with autophagic buildup. Galectin 3, a β-galactoside-binding lectin, belongs to a group of cytosolic galectins that serve as sensors of endosomal-lysosomal damage by virtue of their ability to bind to exposed intraluminal glycans; this binding subsequently leads to autophagic degradation of the damaged organelle.[24,25]

Our data demonstrate a high therapeutic efficacy of AT-GAA. Remarkably, the therapy completely reversed the primary defect—lysosomal glycogen accumulation—and significantly improved a cascade of secondary abnormalities including the elimination of autophagic buildup in >80% of muscle fibers and the restoration (although not complete) of AMPK/mTORC1 signaling and muscle proteostasis. This outcome is all the more striking in light of our early data showing that a long-term treatment (5 months) with the currently available drug—alglucosidase alfa—did not fully correct even the primary defect in KO muscle,[26] not to mention any of the downstream secondary disease mechanisms. However, it appears, that several steps of the pathogenic cascade, particularly those that are removed from the primary defect, such as for example, mTORC1 signaling, are more difficult to reverse. The mechanism of aberrant mTORC1 signaling in the affected muscle is still not fully understood, and the correction of these abnormalities may require a more targeted approach. We and others recently explored the feasibility of mTORC1 reactivation by knocking down TSC2 or by amino acids supplementation in GAA-deficient muscle cells and in KO mice (reviewed in Lim et al.[11]). The lack of further improvement in mTORC1 signaling on long-term versus short-term therapy argues against the possibility that the treatment was not long enough to overcome this signaling abnormality. A more reasonable explanation is that the removal of lysosomal glycogen does not fully restore the composition of phospholipids and cholesterol in the lysosomal membranes, which serve as the sites for mTORC1 activation.[50–52]

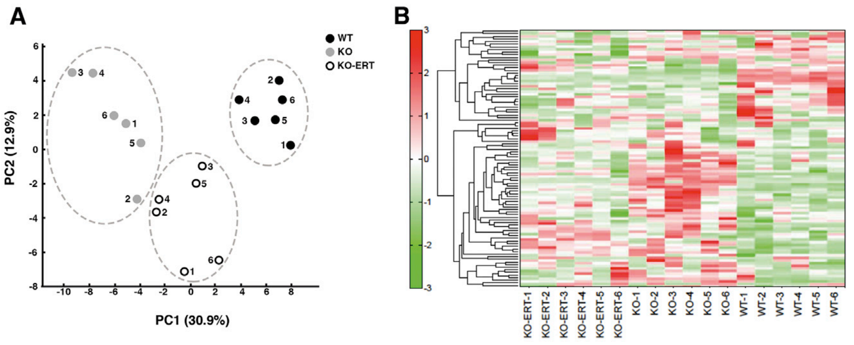

Figure 9. Capillary Electrophoresis-Mass Spectrometry Analysis of Skeletal Muscle from WT, Untreated-, and Treated KO Mice

(A) Principal-component analysis (PCA) of the metabolomic datasets of the skeletal muscle of WT, untreated-, and treated KO mice (n = 6 for each of the three groups). Plots of the three groups are clearly separated on the x axis. (B) A heatmap of hierarchical cluster analysis of the metabolite changes of the skeletal muscle of WT, untreated-, and treated KO mice. The heatmap patterns between WT (6 right lanes) and KO (6 middle three lanes) are clearly distinguishable. The heatmap pattern of treated KO samples (6 left lanes) appears to be closer to that of the WT. Red color indicates relatively high content of metabolites; green indicates relatively low content of metabolites.

In this study, we have also characterized yet another component of the pathogenic cascade in Pompe disease—muscle metabolic alterations. We demonstrate for the first time that there is a shift in energy metabolism pathway from glycolysis to fatty acid β-oxidation in KO muscle, a finding consistent with the shortage of glucose. This conclusion is based on three sets of independent experiments analyzing metabolites in the diseased muscle from young (3–4 months of age), intermediate (5–6 months of age), and old (8–9 months of age) KO mice. A decrease in glycolytic intermediates combined with an increase in carnitine, acetyl-CoA, and TCA cycle intermediates suggest that the affected muscle cells are utilizing fats for energy. As with other elements of the pathogenic cascade, the treatment either reversed or improved the levels of metabolites bringing them closer to the WT values, thus again highlighting the therapeutic efficacy of AT-GAA in Pompe disease.

Disease-related metabolic changes in humans and mice are highly comparable despite the commonly observed differences in the phenotype of many mouse models of human diseases. The same is true for the KO model of Pompe disease. Although the phenotype of KO mice recapitulates all the major characteristics of the disease in humans—generalized glycogen storage, cardiomyopathy, autophagic buildup in skeletal muscle, and neurological deficit—there are some significant differences: despite complete inactivation of the *GAA* gene, the animals, unlike patients, have a much milder disease, they live long enough to grow old and develop obvious clinical signs late, at the age of 7–9 months.[53] However, metabolic pathways are known to be highly conserved through evolution; indeed, metabolic similarities between rodents and humans were observed in several diseases including Alzheimer's disease,[54] Huntington's disease,[55] type 2 diabetes,[56] nonalcoholic fatty liver disease,[57] and colorectal tumors.[58]

Recent study using SeaHorse measurements showed a reduction in glycolysis and in mitochondrial respiration in both patient-derived primary human myoblasts and immortalized GAA-deficient mouse myoblasts.[59] In contrast, metabolome analysis of induced pluripotent stem cell (iPSC)-derived cardiomyocytes from late-onset Pompe disease patients revealed oxidative stress and mitochondrial dysfunction without significant changes in the energy status; no significant differences in the adenylate and guanylate energy charges and in the levels of active AMPK were observed between the diseased and the control iPSC-derived cardiomyocytes.[60] The discrepancy between these two *in vitro* studies may reflect the difference in the metabolic changes in skeletal muscle cells and cardiomyocytes. However, regardless of their origin, the use of cultured cells for metabolomic profile of glycolysis is problematic because the nutrient-rich conditions, which are required for cell culturing, may affect the outcome. For example, activation of glycolytic pathway and a reduction in the Krebs cycle metabolites were observed in renal tubular (HK-2) cells exposed to high glucose.[61]

The notion that glucose deprivation in the diseased muscle may actively contribute to the complex pathogenic cascade in Pompe disease has not been seriously entertained. Perhaps the only notable exception is a publication by Pascual and Row[62] who analyzed blood and urine samples from 33 adult Pompe disease patients treated only with a low-carbohydrate/high-protein diet. The authors presented evidence for disturbed energy metabolism, diminished plasma methylation capacity, and elevated levels of insulin-like growth factor type 1 and suggested that the energy failure due to nutrient sensor disturbance eventually leads to a chronic catabolic state.[62] We hypothesized previously that the lack of lysosome-derived glucose in KO muscle may result in induction of autophagy.[63]

The observed decrease in carbohydrate oxidation and associated increase in fatty acid oxidation for energy supply in KO muscle suggest that the pathophysiology of muscle damage in Pompe disease involves both lysosomal glycogen buildup and a deficit of the recycled glucose. This view fits well with the concept that many lysosomal storage disorders (LSDs) can be defined not only as diseases of excessive lysosomal storage ("overabundance") but also as conditions characterized by the absence/deficiency of recycled precursors leading to a shift in the energy utilization.[64–66] Altered energy balance was described in several LSDs, including mucopolysaccharidosis (MPS) I, IIIB, and VII, Niemann-Pick type A/B, and infantile neuronal ceroid lipofuscinosis (INCL).[64] Metabolic deficiencies of simple sugars, lipids, and nucleotides were found in the liver of MPSI mice, suggesting a state of nutrient deprivation.[65]

Thus, the new finding of a metabolic shift away from glycolysis toward fatty acid oxidation in KO muscle adds to the list of numerous

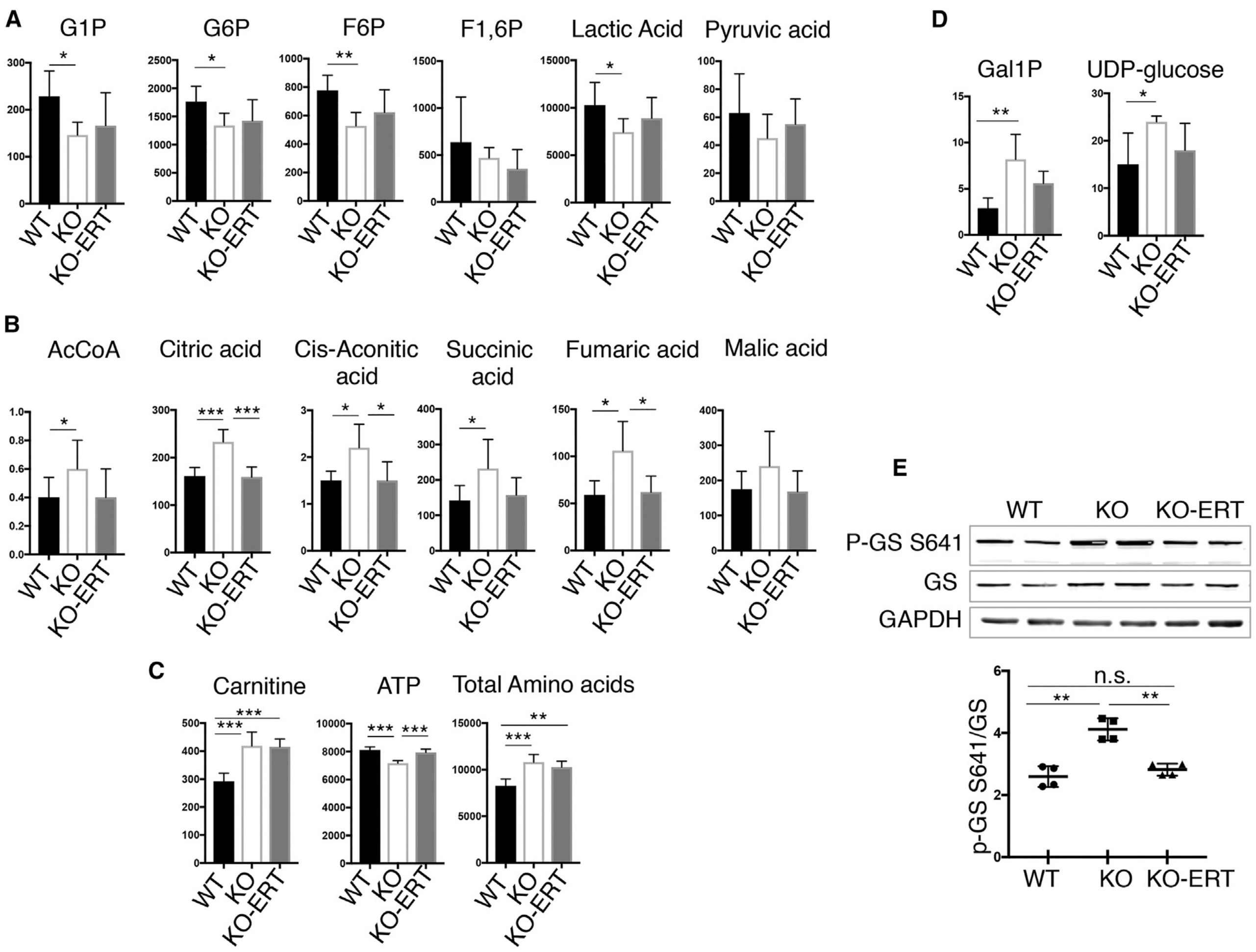

Figure 10. Metabolite Changes in Skeletal Muscle from WT, Untreated-, and Treated KO Mice

Muscle biopsies were collected from age and sex-matched WT, untreated (KO), and AT-GAA-treated KO (KO-ERT) mice. (A–D) The levels of the metabolites involved in the glycolytic pathway (A), TCA cycle (B), carnitine, ATP, and total amino acids (C), and the levels of glycogen precursors (D) in the three groups. (E) Western blot analysis of whole muscle lysates from WT, untreated-, and treated KO mice with indicated antibodies. An increased phosphorylation (inactivation) of glycogen synthase (p-GS S^{641}) in KO muscle (consistent with our previously reported data[10]) is reversed in treated KO mice (n = 4 for each group). Each lane represents a sample from a single mouse. Data are mean ± SD. Statistical significance was determined by one-way ANOVA. *$p < 0.05$; **$p < 0.01$; ***$p < 0.001$.

secondary abnormalities eventually leading to functional impairment in skeletal muscle. The contribution of these defects to the pathophysiology of muscle damage is underscored by early clinical observations in late-onset Pompe disease patients: the severity of myopathy was disproportionate to the relatively modest glycogen accumulation in muscle biopsies.[67] Similarly, a relatively slow rate of glycogen buildup in skeletal muscle of KO mice was demonstrated by us and others.[26,68]

Lysosomal glycogen degradation by glycogen-hydrolyzing GAA triggers liberation of non-phosphorylated glucose, whereas cytosolic glycogen is broken down to glucose-1-phosphate via glycogenolysis. One of the unanswered questions is how these two processes communicate with each other. Recent studies identified several proteins that can serve as sensors of glucose availability. The glycolytic enzyme aldolase was shown to activate AMPK during early phase of glucose deprivation (before changes in the cellular AMP:ATP ratio develop) due to the inability of the enzyme to bind fructose-1,6-bisphosphate, when the concentration of this glycolytic intermediate drops.[69] Hexokinase-II was implicated in autophagy induction following glucose deprivation by virtue of binding and inhibiting mTORC1 in cardiomyocyte and muscle cells.[70] Another example is glycogen synthase, which was proposed to function as a sensor of glucose released by lysosomal glycogen degradation.[71] It stands to reason that a closer look at these processes in Pompe disease can be rewarding.

In conclusion, we have expanded our understanding of the downstream disease mechanisms in Pompe disease and demonstrated that the next generation recombinant enzyme, AT-GAA, can correct or improve multiple defects along the pathogenic cascade. Our findings also indicate that some of the downstream pathologic changes

still persist even though the clearance of accumulated glycogen appears to be complete. Finally, this study sets standards for rigorous testing of new therapies.

MATERIALS AND METHODS

Antibodies

The following antibodies were used: total and phosphorylated S6 (S235/236; #2217, #4858; rabbit monoclonal), total and phosphorylated AKT (S473; #4691, #4060; rabbit monoclonal), total and phosphorylated PRAS40 (T246; #2691, #2997; rabbit monoclonal), total and phosphorylated eIF2 alpha (S51; #9722, #3398; rabbit polyclonal and monoclonal, respectively), total and phosphorylated TSC2 (S1387; #4308, #5584; rabbit polyclonal and monoclonal, respectively), total and phosphorylated ULK1 (S317; #8054, #6887; rabbit monoclonal and polyclonal, respectively), total and phosphorylated acetyl-CoA-carboxylase (S79; #3676, #11818; rabbit monoclonal), total and phosphorylated TBC1D1 (S660; #5929, #6928; rabbit monoclonal and polyclonal, respectively), total and phosphorylated (T172; #5831, #2535; rabbit monoclonal), total and non-phosphorylated 4EBP1 (T46; #9644, #4923; rabbit monoclonal), phosphorylated 4EBP1 (T37/46 and S65; #9459 and #9451; rabbit polyclonal), total and phosphorylated glycogen synthase (S641; #3886, #3891; rabbit monoclonal and polyclonal, respectively), ATG7 (#2631; rabbit polyclonal), and LKB1 (#3047; rabbit monoclonal); all were purchased from Cell Signaling Technology (1:1,000 dilution). Beclin (#612112; mouse monoclonal), Rab5 (#610724; mouse monoclonal), and LAMP1 (CD107a #553792; rat monoclonal) were purchased from BD Transduction Laboratories. SQSTM1/p62 (#ab56416; mouse monoclonal), galectin 9 (#ab69630; rabbit polyclonal), Ubiquitin K48 (Linkage Specific) (#ab140601; rabbit monoclonal), and glyceraldehyde-3-phosphate dehydrogenase (#ab9485; rabbit polyclonal) were purchased from Abcam. Galectin 3 (#sc-32790; mouse monoclonal) were from Santa Cruz Biotechnology; Grp78/BiP (HspA5; #ADI-SPA-826; rabbit polyclonal) were from ENZO. Ubiquitin K63 (#05-1308; rabbit monoclonal), Vinculin (#V9131; mouse monoclonal), and LC3B (#L7543; rabbit polyclonal) were purchased from Sigma Aldrich. VPS-34 (#NB110-87320; rabbit polyclonal) and PIK3R4 (#NBP1-30463; rabbit polyclonal) were purchased from Novus Biologicals. GLUT4 (#GT41-A; rabbit polyclonal) were from Alpha Diagnostic International.

Animal Models, Treatment, and Muscle Tissue Processing

GAA KO mice (a mouse model of Pompe disease[53]) were used for the experiments. Muscle-specific autophagy-deficient $GAA^{-/-}$ mice (MLCcre: Atg7F/F: $GAA^{-/-}$; referred to as DKO[27]) were used for the evaluation of galectin 3 levels in skeletal muscle. In these DKO mice, a critical autophagy gene, ATG7, was excised in skeletal muscle by Cre recombinase. Female KO mice (at the ages of 3–4 months; n = 6) received 11 biweekly tail vein i.v. injections of ATB200 (Amicus proprietary rhGAA; 20 mg/kg) in combination with the pharmacological chaperone AT2221 (miglustat, N-butyl-deoxynojirimycin;10 mg/kg). Age- and sex-matched WT and untreated KO mice were used for the experiments. This group of mice was used to prepare muscle tissue for western blotting, for isolation of single muscle fibers (from one hind limb), and for metabolome analysis (from the other hind limb). The second set of WT untreated- and AT-GAA-treated (the dosage, regimen, and the age at start of therapy were as described above) male mice (n = 3 for each group) was used for the puromycin experiments. An additional third set (n = 3 for each group) was used to obtain muscle samples for isolation of proteasome-enriched fraction and evaluation of proteasomal activity. To prevent anaphylaxis, we injected diphenhydramine intraperitoneally at a dose of 10 mg/kg 15 min prior to the second AT-GAA administration. Total number of injections were 11–12, and the animals were sacrificed 7–10 days after the last injection. In addition, WT and untreated KO mice of different ages (10-day-old, 6-week-old, 3-month-old) were used for galectin 3 measurement in different tissues and serum and for immunostaining of single muscle fibers with autophagosomal/lysosomal markers (at least three independent experiments were done for each analysis). In all experiments, the white part of gastrocnemius muscle was excised. Parts of the biopsy were frozen in isopentane chilled in liquid nitrogen and stored at $-80^{\circ}C$ until analysis. Parts of the biopsy were fixed in 2% paraformaldehyde for immunostaining of single muscle fibers and SHG imaging.

Measurement of GAA Activity and Glycogen Levels

A fluorescence assay using 4-methylumbelliferyl-α-D-glucoside as the fluorogenic GAA substrate was employed for measuring GAA activity as described.[72] Briefly, muscle samples were homogenized in distilled water, sonicated, and centrifuged at 18,000 × g at 4°C for 15 min. The supernatants were incubated with the substrate in 0.2 M sodium acetate buffer (pH 4.3) for 1 h at 37°C; the reaction was stopped by adding 0.5 M carbonate buffer (pH 10.5). 4-Methylumbelliferone (Sigma-Aldrich) was used as a standard. Fluorescence was measured on a multi-label plate reader (TECAN, SPARK 10M) at 360 nm excitation/465 nm emission.

Glycogen content in tissue homogenates was measured as the amount of glucose released after glycogen digestion with Aspergillus Niger amyloglucosidase (Sigma-Aldrich). Samples were boiled for 3 min to inactivate endogenous enzymes, incubated with or without 0.175 U/mL amyloglucosidase for 90 min at 37°C in 0.1 M potassium acetate buffer (pH 5.5) and boiled again to stop the reaction. The released glucose was measured using Glucose (Hexokinase) Liquid Reagents (Fisher) as recommended in the instructions; the absorbance at 340 nm was read on the Agilent Technologies Cary 60 UV-VIS Spectrophotometer. Protein concentration was determined by BCA assay and used to normalize the data.

Functional Muscle Strength Test

Muscle strength test was performed using a Grip Strength Meter (Columbus Instruments) according to the instructions recommended by the manufacturer. The test measures maximal forelimbs/hindlimbs grip strength as an indicator of neuromuscular function. The animals are allowed to grasp the grid (that is connected to a sensor) and are then pulled backward in the horizontal plane. The force applied to the grid is recorded as the maximal peak force displayed by an animal.

The tests were performed after 8 and 9 administrations of AT-GAA (n = 5 WT; n = 4 KO; n = 7 KO-ERT). Each mouse was subjected to 3 trials in one day, and the trials were carried out on 3 consecutive days.

Western Blot Analysis

For total protein isolation, the white part of gastrocnemius muscle (n = 4–6 from each category) was homogenized in radioimmunoprecipitation assay (RIPA) buffer (PBS containing 1% NP40, 0.5% sodium deoxycholate, 0.1% SDS, and a protease/phosphatase inhibitor cocktail (#5872, Cell Signaling Technology). Total protein lysates were centrifuged for 15 min at 16,000 $\times$ *g* at 4°C. Proteins were separated by SDS-PAGE gels (Invitrogen, Carlsbad, CA, USA) by loading equal amount. Separated proteins were transferred onto nitrocellulose membranes (Invitrogen, Carlsbad, CA, USA). Membranes were then treated with blocking buffer (5% nonfat milk), incubated with primary antibodies, washed and incubated with the appropriate secondary antibodies. Horseradish peroxidase (HRP)-chemiluminescence was developed using Azure Radiance plus kit and scanned on imager (Azure Biosystems). Alternatively, membranes were treated with Odyssey Blocking Buffer (LI-COR Biosciences) and incubated overnight with primary antibodies. After washing, membranes were incubated with fluorophore conjugated secondary antibodies and scanned on an infrared imager (LI-COR Biosciences).

Measuring the Rate of Protein Synthesis and Proteasome Activity

SUnSET method was used to measure protein synthesis rate as described.[44] WT, untreated-, and treated KO mice (n = 3 for each group) received a single puromycin (Invitrogen; #A11138-03) injection intraperitonially at a dose of 0.04 μmol/g, 30 min before sacrifice. The amount of puromycin incorporated into nascent peptides was evaluated by immunoblot using anti-puromycin antibody. Method for proteasome-enrichment was adapted from Hobler et al.[73] Briefly, gastrocnemius muscles were homogenized in ice cold buffer (pH 7.5) containing $MgCl_2$ (5 mM), Tris-HCL (50 mM), and sucrose (250 mM). The lysates were first centrifuged at 16,000 $\times$ *g* for 20 min, and the supernatants were subjected to two sequential centrifugation at 100,000 $\times$ *g* for 1 hr and 150,000 $\times$ *g* for 5 hr. Proteasome-enriched pellet was resuspended in ice cold buffer containing Tris (40 mM), NaCl (50 mM), mercaptoethanol (2 mM), $MgCl_2$ (5 mM), and glycerol (10%). Proteasome activity was measured using Proteasome Activity Fluorometric Assay Kit II (UBPBio, Aurora, CO, USA; #J4120) as recommended by the manufacturer. Fluorogenic peptide substrates, Z-LLE-AMC, Suc-LLVY-AMC, and Boc-LRR-AMC, were used for caspase-, chymotrypsin-, and trypsin-like activities, respectively.

Immunostaining of Single Muscle Fibers, SHG Microscopy, and Measurement of Galectin 3 in Serum

Muscle fixation, isolation of single fibers, and immunostaining were described in detail.[74] Four mice from each group (WT, untreated-, and treated KO) were used to obtain single muscle fibers for immunostaining. For each immunostaining and for confocal analysis, at least 50 fibers were isolated. The images were captured using a Zeiss LSM 780 confocal microscope under the Zeiss Efficient Navigation (ZEN) software. Muscle samples for SHG microscopy were fixed with 2% paraformaldehyde (Electron Microscopy Science) in 0.1 M phosphate buffer, cut longitudinally into bundles of 200–300 μm thickness, mounted in a chamber filled with 50% glycerol and sealed with glass coverslips. For longer storage, muscle bundles were transferred to 12.5%, then 25% and 50% glycerol in phosphate-buffered saline (PBS) and stored at −20°C. SHG microscopy was performed as described previously[23] on a Leica SP5 NLO confocal microscope equipped with a Mai Tai two-photon laser (Newport/Spectra-Physics). Galectin 3 in mouse serum was measured using *in vitro* SimpleStep ELISA (enzyme-linked immunosorbent assay) kit (Abcam, ab203369) according to the manufacturer's instructions.

Metabolome Analysis

The white part of gastrocnemius muscles was excised from WT, untreated-, and treated KO mice (n = 6 in each category). Muscle tissues were immediately frozen in isopentane (2 methylbutane) chilled with liquid N_2 and stored at −80°C. Muscle metabolome was determined by capillary electrophoresis time of flight mass spectrometer (CE-TOF/MS) and capillary electrophoresis-triple quadrupole mass spectrometry (CE-QqQMS) in the cation and anion analysis modes; the analysis was performed by Human Metabolome Technologies (HMT, Tsuruoka, Japan). Metabolite peaks were quantified and annotated based on an HMT metabolite database (http://humanmetabolome.com/).

Statistics

Statistical significance was calculated by using GraphPad Prism software. One-way ANOVA and unpaired two-tailed Student's t test were performed. Data presented as mean ± SD; *p < 0.05; **p < 0.01; ***p < 0.001; ****p < 0.0001).

Study Approval

Animal care and experiments were conducted in accordance with the National Institutes of Health Guide for the Care and Use of Laboratory Animals.

SUPPLEMENTAL INFORMATION

Supplemental Information can be found online at https://doi.org/10.1016/j.omtm.2020.05.026.

AUTHOR CONTRIBUTIONS

N.K.M. and E.R. performed experiments, analyzed and interpreted the data and participated in preparation of the manuscript; N.R. and R.P. designed, interpreted, and analyzed data and wrote the paper.

CONFLICTS OF INTEREST

The authors declare no competing interests.

ACKNOWLEDGMENTS

We are grateful to our colleagues at Amicus Therapeutics for providing AT-GAA and for helpful discussions. We would like to

thank Davide Randazzo (Light Imaging Section, Office of Science and Technology, NIAMS, NIH) for his help with the imaging. This research was supported by the Intramural Research Program of the NHLBI of the National Institutes of Health. N.K.M. was supported in part by a CRADA between NIH and Amicus Therapeutics.

REFERENCES

1. Barton, N.W., Brady, R.O., Dambrosia, J.M., Di Bisceglie, A.M., Doppelt, S.H., Hill, S.C., Mankin, H.J., Murray, G.J., Parker, R.I., Argoff, C.E., et al. (1991). Replacement therapy for inherited enzyme deficiency–macrophage-targeted glucocerebrosidase for Gaucher's disease. N. Engl. J. Med. *324*, 1464–1470.

2. Engel, A.G., Hirschorn, R., and Huie, M.L. (2003). Acid Maltase Deficiency. In Myology, A.G. Engel and C. Franzini-Armstrong C. eds. (New York McGraw-Hill), pp. 1559–1586.

3. van der Ploeg, A.T., and Reuser, A.J. (2008). Pompe's disease. Lancet *372*, 1342–1353.

4. Prater, S.N., Banugaria, S.G., DeArmey, S.M., Botha, E.G., Stege, E.M., Case, L.E., Jones, H.N., Phornphutkul, C., Wang, R.Y., Young, S.P., and Kishnani, P.S. (2012). The emerging phenotype of long-term survivors with infantile Pompe disease. Genet. Med. *14*, 800–810.

5. Hahn, A., and Schänzer, A. (2019). Long-term outcome and unmet needs in infantile-onset Pompe disease. Ann. Transl. Med. *7*, 283.

6. Chan, J., Desai, A.K., Kazi, Z.B., Corey, K., Austin, S., Hobson-Webb, L.D., Case, L.E., Jones, H.N., and Kishnani, P.S. (2017). The emerging phenotype of late-onset Pompe disease: A systematic literature review. Mol. Genet. Metab. *120*, 163–172.

7. Toscano, A., Rodolico, C., and Musumeci, O. (2019). Multisystem late onset Pompe disease (LOPD): an update on clinical aspects. Ann. Transl. Med. *7*, 284.

8. van Kooten, H.A., Harlaar, L., van der Beek, N., van Doorn, P.A., van der Ploeg, A.T., Brusse, E., et al. (2019). Discontinuation of enzyme replacement therapy in adults with Pompe disease: Evaluating the European POmpe Consortium stop criteria. Neuromuscul. Disord. *30*, 59–66.

9. Kohler, L., Puertollano, R., and Raben, N. (2018). Pompe Disease: From Basic Science to Therapy. Neurotherapeutics *15*, 928–942.

10. Xu, S., Lun, Y., Frascella, M., Garcia, A., Soska, R., Nair, A., Ponery, A.S., Schilling, A., Feng, J., Tuske, S., et al. (2019). Improved efficacy of a next-generation ERT in murine Pompe disease. JCI Insight *4*, e125358.

11. Lim, J.A., Meena, N.K., and Raben, N. (2019). Pros and cons of different ways to address dysfunctional autophagy in Pompe disease. Ann. Transl. Med. *7*, 279.

12. Lim, J.A., Li, L., Shirihai, O.S., Trudeau, K.M., Puertollano, R., and Raben, N. (2017). Modulation of mTOR signaling as a strategy for the treatment of Pompe disease. EMBO Mol. Med. *9*, 353–370.

13. Lim, J.A., Sun, B., Puertollano, R., and Raben, N. (2018). Therapeutic Benefit of Autophagy Modulation in Pompe Disease. Mol. Ther. *26*, 1783–1796.

14. Stjepanovic, G., Baskaran, S., Lin, M.G., and Hurley, J.H. (2017). Unveiling the role of VPS34 kinase domain dynamics in regulation of the autophagic PI3K complex. Mol. Cell. Oncol. *4*, e1367873.

15. Stjepanovic, G., Baskaran, S., Lin, M.G., and Hurley, J.H. (2017). Vps34 Kinase Domain Dynamics Regulate the Autophagic PI 3-Kinase Complex. Mol. Cell *67*, 528–534.

16. Nascimbeni, A.C., Fanin, M., Angelini, C., and Sandri, M. (2017). Autophagy dysregulation in Danon disease. Cell Death Dis. *8*, e2565.

17. Komatsu, M., Tanida, I., Ueno, T., Ohsumi, M., Ohsumi, Y., and Kominami, E. (2001). The C-terminal region of an Apg7p/Cvt2p is required for homodimerization and is essential for its E1 activity and E1-E2 complex formation. J. Biol. Chem. *276*, 9846–9854.

18. Ichimura, Y., Kirisako, T., Takao, T., Satomi, Y., Shimonishi, Y., Ishihara, N., Mizushima, N., Tanida, I., Kominami, E., Ohsumi, M., et al. (2000). A ubiquitin-like system mediates protein lipidation. Nature *408*, 488–492.

19. Bjørkøy, G., Lamark, T., Brech, A., Outzen, H., Perander, M., Overvatn, A., Stenmark, H., and Johansen, T. (2005). p62/SQSTM1 forms protein aggregates degraded by autophagy and has a protective effect on huntingtin-induced cell death. J. Cell Biol. *171*, 603–614.

20. Kabeya, Y., Mizushima, N., Ueno, T., Yamamoto, A., Kirisako, T., Noda, T., Kominami, E., Ohsumi, Y., and Yoshimori, T. (2000). LC3, a mammalian homologue of yeast Apg8p, is localized in autophagosome membranes after processing. EMBO J. *19*, 5720–5728.

21. Campagnola, P.J., and Loew, L.M. (2003). Second-harmonic imaging microscopy for visualizing biomolecular arrays in cells, tissues and organisms. Nat. Biotechnol. *21*, 1356–1360.

22. Plotnikov, S.V., Millard, A.C., Campagnola, P.J., and Mohler, W.A. (2006). Characterization of the myosin-based source for second-harmonic generation from muscle sarcomeres. Biophys. J. *90*, 693–703.

23. Ralston, E., Swaim, B., Czapiga, M., Hwu, W.L., Chien, Y.H., Pittis, M.G., Bembi, B., Schwartz, O., Plotz, P., and Raben, N. (2008). Detection and imaging of non-contractile inclusions and sarcomeric anomalies in skeletal muscle by second harmonic generation combined with two-photon excited fluorescence. J. Struct. Biol. *162*, 500–508.

24. Papadopoulos, C., and Meyer, H. (2017). Detection and Clearance of Damaged Lysosomes by the Endo-Lysosomal Damage Response and Lysophagy. Curr. Biol. *27*, R1330–R1341.

25. Papadopoulos, C., Kravic, B., and Meyer, H. (2020). Repair or Lysophagy: Dealing with Damaged Lysosomes. J. Mol. Biol. *432*, 231–239.

26. Raben, N., Danon, M., Gilbert, A.L., Dwivedi, S., Collins, B., Thurberg, B.L., Mattaliano, R.J., Nagaraju, K., and Plotz, P.H. (2003). Enzyme replacement therapy in the mouse model of Pompe disease. Mol. Genet. Metab. *80*, 159–169.

27. Raben, N., Schreiner, C., Baum, R., Takikita, S., Xu, S., Xie, T., Myerowitz, R., Komatsu, M., Van der Meulen, J.H., Nagaraju, K., et al. (2010). Suppression of autophagy permits successful enzyme replacement therapy in a lysosomal storage disorder–murine Pompe disease. Autophagy *6*, 1078–1089.

28. Taylor, E.B., An, D., Kramer, H.F., Yu, H., Fujii, N.L., Roeckl, K.S., Bowles, N., Hirshman, M.F., Xie, J., Feener, E.P., and Goodyear, L.J. (2008). Discovery of TBC1D1 as an insulin-, AICAR-, and contraction-stimulated signaling nexus in mouse skeletal muscle. J. Biol. Chem. *283*, 9787–9796.

29. Szekeres, F., Chadt, A., Tom, R.Z., Deshmukh, A.S., Chibalin, A.V., Björnholm, M., Al-Hasani, H., and Zierath, J.R. (2012). The Rab-GTPase-activating protein TBC1D1 regulates skeletal muscle glucose metabolism. Am. J. Physiol. Endocrinol. Metab. *303*, E524–E533.

30. Cartee, G.D. (2015). Roles of TBC1D1 and TBC1D4 in insulin- and exercise-stimulated glucose transport of skeletal muscle. Diabetologia *58*, 19–30.

31. Douillard-Guilloux, G., Raben, N., Takikita, S., Ferry, A., Vignaud, A., Guillet-Deniau, I., Favier, M., Thurberg, B.L., Roach, P.J., Caillaud, C., and Richard, E. (2010). Restoration of muscle functionality by genetic suppression of glycogen synthesis in a murine model of Pompe disease. Hum. Mol. Genet. *19*, 684–696.

32. Inoki, K., Kim, J., and Guan, K.L. (2012). AMPK and mTOR in cellular energy homeostasis and drug targets. Annu. Rev. Pharmacol. Toxicol. *52*, 381–400.

33. Egan, D., Kim, J., Shaw, R.J., and Guan, K.L. (2011). The autophagy initiating kinase ULK1 is regulated via opposing phosphorylation by AMPK and mTOR. Autophagy *7*, 643–644.

34. Kim, J., Kundu, M., Viollet, B., and Guan, K.L. (2011). AMPK and mTOR regulate autophagy through direct phosphorylation of Ulk1. Nat. Cell Biol. *13*, 132–141.

35. Sengupta, S., Peterson, T.R., and Sabatini, D.M. (2010). Regulation of the mTOR complex 1 pathway by nutrients, growth factors, and stress. Mol. Cell *40*, 310–322.

36. Carroll, B., and Dunlop, E.A. (2017). The lysosome: a crucial hub for AMPK and mTORC1 signalling. Biochem. J. *474*, 1453–1466.

37. Inoki, K., Li, Y., Xu, T., and Guan, K.L. (2003). Rheb GTPase is a direct target of TSC2 GAP activity and regulates mTOR signaling. Genes Dev. *17*, 1829–1834.

38. Benjamin, D., and Hall, M.N. (2014). mTORC1: turning off is just as important as turning on. Cell *156*, 627–628.

39. Menon, S., Dibble, C.C., Talbott, G., Hoxhaj, G., Valvezan, A.J., Takahashi, H., Cantley, L.C., and Manning, B.D. (2014). Spatial control of the TSC complex integrates insulin and nutrient regulation of mTORC1 at the lysosome. Cell *156*, 771–785.

40. Laplante, M., and Sabatini, D.M. (2012). mTOR signaling in growth control and disease. Cell *149*, 274–293.

41. Sancak, Y., Thoreen, C.C., Peterson, T.R., Lindquist, R.A., Kang, S.A., Spooner, E., Carr, S.A., and Sabatini, D.M. (2007). PRAS40 is an insulin-regulated inhibitor of the mTORC1 protein kinase. Mol. Cell *25*, 903–915.

42. Hay, N., and Sonenberg, N. (2004). Upstream and downstream of mTOR. Genes Dev. *18*, 1926–1945.

43. Aitken, C.E., and Lorsch, J.R. (2012). A mechanistic overview of translation initiation in eukaryotes. Nat. Struct. Mol. Biol. *19*, 568–576.

44. Goodman, C.A., Mabrey, D.M., Frey, J.W., Miu, M.H., Schmidt, E.K., Pierre, P., and Hornberger, T.A. (2011). Novel insights into the regulation of skeletal muscle protein synthesis as revealed by a new nonradioactive in vivo technique. FASEB J. *25*, 1028–1039.

45. Lim, J.A., Li, L., Kakhlon, O., Myerowitz, R., and Raben, N. (2015). Defects in calcium homeostasis and mitochondria can be reversed in Pompe disease. Autophagy *11*, 385–402.

46. Murthy, M.S., and Pande, S.V. (1984). Mechanism of carnitine acylcarnitine translocase-catalyzed import of acylcarnitines into mitochondria. J. Biol. Chem. *259*, 9082–9089.

47. Settembre, C., Fraldi, A., Medina, D.L., and Ballabio, A. (2013). Signals from the lysosome: a control centre for cellular clearance and energy metabolism. Nat. Rev. Mol. Cell Biol. *14*, 283–296.

48. Efeyan, A., Comb, W.C., and Sabatini, D.M. (2015). Nutrient-sensing mechanisms and pathways. Nature *517*, 302–310.

49. Perera, R.M., and Zoncu, R. (2016). The Lysosome as a Regulatory Hub. Annu. Rev. Cell Dev. Biol. *32*, 223–253.

50. Sancak, Y., Bar-Peled, L., Zoncu, R., Markhard, A.L., Nada, S., and Sabatini, D.M. (2010). Ragulator-Rag complex targets mTORC1 to the lysosomal surface and is necessary for its activation by amino acids. Cell *141*, 290–303.

51. Zoncu, R., Bar-Peled, L., Efeyan, A., Wang, S., Sancak, Y., and Sabatini, D.M. (2011). mTORC1 senses lysosomal amino acids through an inside-out mechanism that requires the vacuolar H(+)-ATPase. Science *334*, 678–683.

52. Bar-Peled, L., and Sabatini, D.M. (2014). Regulation of mTORC1 by amino acids. Trends Cell Biol. *24*, 400–406.

53. Raben, N., Nagaraju, K., Lee, E., Kessler, P., Byrne, B., Lee, L., LaMarca, M., King, C., Ward, J., Sauer, B., and Plotz, P. (1998). Targeted disruption of the acid alpha-glucosidase gene in mice causes an illness with critical features of both infantile and adult human glycogen storage disease type II. J. Biol. Chem. *273*, 19086–19092.

54. Trushina, E., and Mielke, M.M. (2014). Recent advances in the application of metabolomics to Alzheimer's Disease. Biochim. Biophys. Acta *1842*, 1232–1239.

55. Underwood, B.R., Broadhurst, D., Dunn, W.B., Ellis, D.I., Michell, A.W., Vacher, C., Mosedale, D.E., Kell, D.B., Barker, R.A., Grainger, D.J., and Rubinsztein, D.C. (2006). Huntington disease patients and transgenic mice have similar pro-catabolic serum metabolite profiles. Brain *129*, 877–886.

56. Salek, R.M., Maguire, M.L., Bentley, E., Rubtsov, D.V., Hough, T., Cheeseman, M., Nunez, D., Sweatman, B.C., Haselden, J.N., Cox, R.D., et al. (2007). A metabolomic comparison of urinary changes in type 2 diabetes in mouse, rat, and human. Physiol. Genomics *29*, 99–108.

57. Barr, J., Vázquez-Chantada, M., Alonso, C., Pérez-Cormenzana, M., Mayo, R., Galán, A., Caballería, J., Martín-Duce, A., Tran, A., Wagner, C., et al. (2010). Liquid chromatography-mass spectrometry-based parallel metabolic profiling of human and mouse model serum reveals putative biomarkers associated with the progression of nonalcoholic fatty liver disease. J. Proteome Res. *9*, 4501–4512.

58. Manna, S.K., Tanaka, N., Krausz, K.W., Haznadar, M., Xue, X., Matsubara, T., Bowman, E.D., Fearon, E.R., Harris, C.C., Shah, Y.M., and Gonzalez, F.J. (2014). Biomarkers of coordinate metabolic reprogramming in colorectal tumors in mice and humans. Gastroenterology *146*, 1313–1324.

59. Meinke, P., Limmer, S., Hintze, S., and Schoser, B. (2019). Assessing metabolic profiles in human myoblasts from patients with late-onset Pompe disease. Ann. Transl. Med. *7*, 277.

60. Sato, Y., Kobayashi, H., Higuchi, T., Shimada, Y., Ida, H., and Ohashi, T. (2017). Metabolomic Profiling of Pompe Disease-Induced Pluripotent Stem Cell-Derived Cardiomyocytes Reveals That Oxidative Stress Is Associated with Cardiac and Skeletal Muscle Pathology. Stem Cells Transl. Med. *6*, 31–39.

61. Wei, P.Z., Fung, W.W., Ng, J.K., Lai, K.B., Luk, C.C., Chow, K.M., Li, P.K., and Szeto, C.C. (2019). Metabolomic Changes of Human Proximal Tubular Cell Line in High Glucose Environment. Sci. Rep. *9*, 16617.

62. Pascual, J.M., and Roe, C.R. (2013). Systemic metabolic abnormalities in adult-onset acid maltase deficiency: beyond muscle glycogen accumulation. JAMA Neurol. *70*, 756–763.

63. Fukuda, T., Ewan, L., Bauer, M., Mattaliano, R.J., Zaal, K., Ralston, E., Plotz, P.H., and Raben, N. (2006). Dysfunction of endocytic and autophagic pathways in a lysosomal storage disease. Ann. Neurol. *59*, 700–708.

64. Woloszynek, J.C., Coleman, T., Semenkovich, C.F., and Sands, M.S. (2007). Lysosomal dysfunction results in altered energy balance. J. Biol. Chem. *282*, 35765–35771.

65. Woloszynek, J.C., Kovacs, A., Ohlemiller, K.K., Roberts, M., and Sands, M.S. (2009). Metabolic adaptations to interrupted glycosaminoglycan recycling. J. Biol. Chem. *284*, 29684–29691.

66. Walkley, S.U. (2009). Pathogenic cascades in lysosomal disease-Why so complex? J. Inherit. Metab. Dis. *32*, 181–189.

67. Slonim, A.E., Coleman, R.A., McElligot, M.A., Najjar, J., Hirschhorn, K., Labadie, G.U., Mrak, R., Evans, O.B., Shipp, E., and Presson, R. (1983). Improvement of muscle function in acid maltase deficiency by high-protein therapy. Neurology *33*, 34–38.

68. Lagalice, L., Pichon, J., Gougeon, E., Soussi, S., Deniaud, J., Ledevin, M., Maurier, V., Leroux, I., Durand, S., Ciron, C., et al. (2018). Satellite cells fail to contribute to muscle repair but are functional in Pompe disease (glycogenosis type II). Acta Neuropathol. Commun. *6*, 116.

69. Zhang, C.S., Hawley, S.A., Zong, Y., Li, M., Wang, Z., Gray, A., Ma, T., Cui, J., Feng, J.W., Zhu, M., et al. (2017). Fructose-1,6-bisphosphate and aldolase mediate glucose sensing by AMPK. Nature *548*, 112–116.

70. Roberts, D.J., Tan-Sah, V.P., Ding, E.Y., Smith, J.M., and Miyamoto, S. (2014). Hexokinase-II positively regulates glucose starvation-induced autophagy through TORC1 inhibition. Mol. Cell *53*, 521–533.

71. Zirin, J., Nieuwenhuis, J., and Perrimon, N. (2013). Role of autophagy in glycogen breakdown and its relevance to chloroquine myopathy. PLoS Biol. *11*, e1001708.

72. Amalfitano, A., McVie-Wylie, A.J., Hu, H., Dawson, T.L., Raben, N., Plotz, P., and Chen, Y.T. (1999). Systemic correction of the muscle disorder glycogen storage disease type II after hepatic targeting of a modified adenovirus vector encoding human acid-alpha-glucosidase. Proc. Natl. Acad. Sci. USA *96*, 8861–8866.

73. Hobler, S.C., Williams, A., Fischer, D., Wang, J.J., Sun, X., Fischer, J.E., Monaco, J.J., and Hasselgren, P.O. (1999). Activity and expression of the 20S proteasome are increased in skeletal muscle during sepsis. Am. J. Physiol. *277*, R434–R440.

74. Raben, N., Shea, L., Hill, V., and Plotz, P. (2009). Monitoring autophagy in lysosomal storage disorders. Methods Enzymol. *453*, 417–449.

OMTM, Volume *18*

Supplemental Information

Enzyme Replacement Therapy Can Reverse Pathogenic Cascade in Pompe Disease

Naresh Kumar Meena, Evelyn Ralston, Nina Raben, and Rosa Puertollano

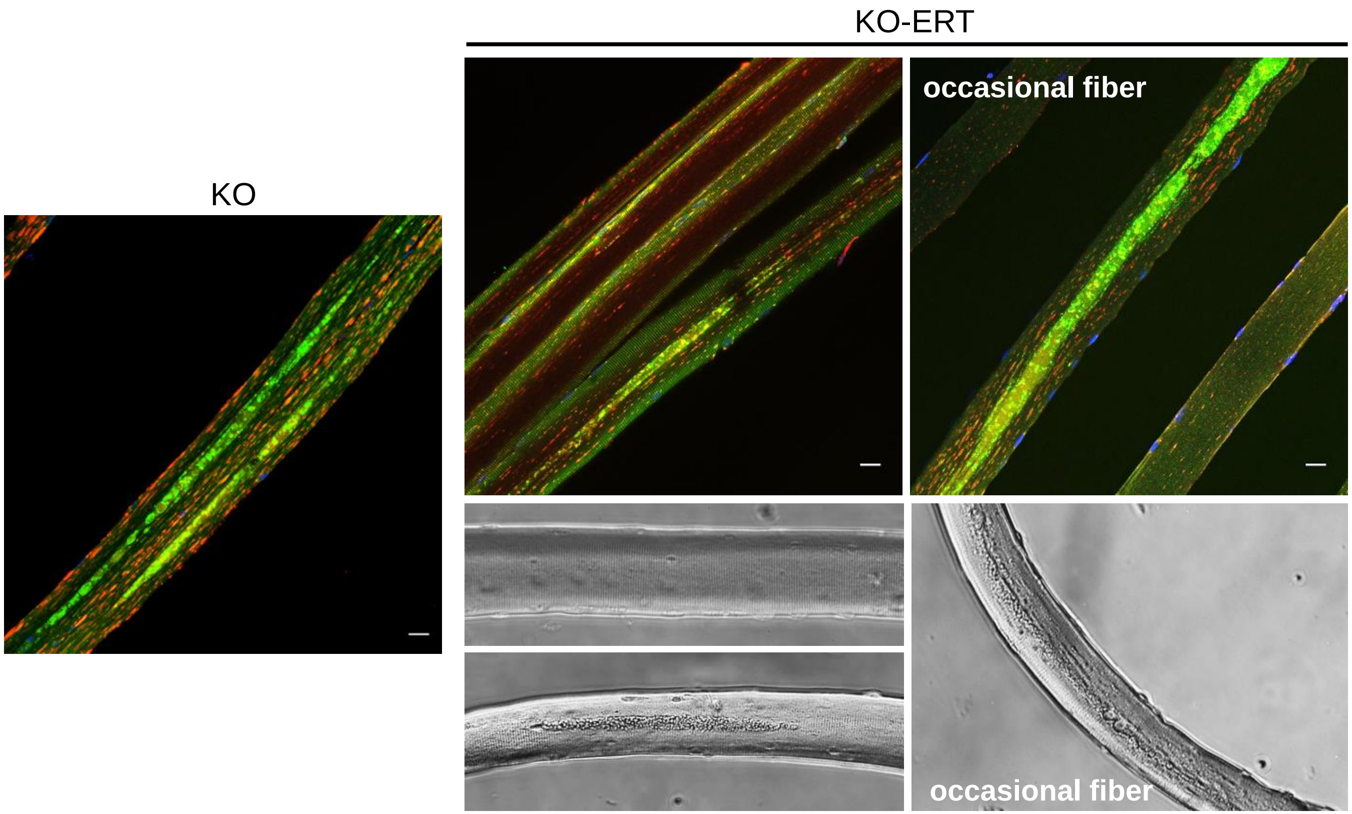

Figure S1. Additional images of muscle fibers from untreated- and treated KO mice.

Confocal microscopy images of single muscle fibers immunostained with lysosomal marker LAMP1 (red) and autophagosomal marker LC3 (green); nuclei are stained with Hoechst dye (blue). Virtually all muscle fibers (the white part of gastrocnemius) from 9-month-old untreated KO contain large areas of autophagic buildup often spanning the whole length of the fibers. Most fibers from 9-month-old treated KO mice (the animals received 11 bi-weekly i.v. administrations of AT-GAA as described in the main text) are free from autophagic buildup, whereas ~16% fibers contain autophagic buildup of smaller size, and only occasional fibers contain autophagic buildup of the size as the one seen in untreated KO mice. Autophagic buildup can also be detected by bright-field microscopy or differential interference contrast microscopy. Bar: 20 µm.

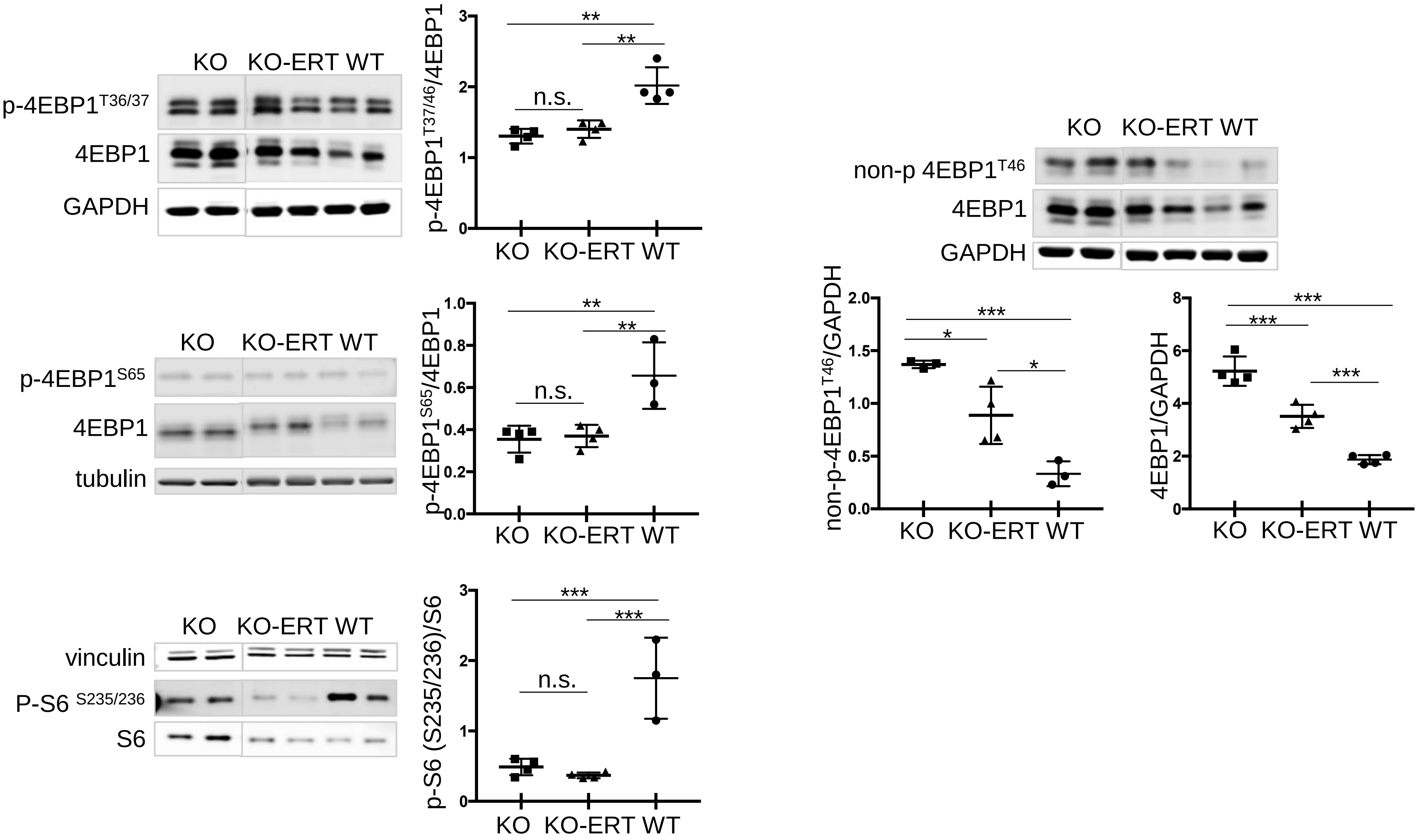

Figure S2. The effect of AT-GAA on mTORC1 signaling following a short-term regimen. KO mice (3-4- month-old) received 4 bi-weekly i.v. administrations of AT-GAA as described in the main text. Age and sex-matched WT and untreated KO mice were used for the comparisons. Skeletal muscle biopsies (the white part of gastrocnemius) were collected 14 days after the last administration. Western blot analysis of muscle lysates from WT, untreated KO (KO), and treated KO (KO-ERT) mice with indicated antibodies (n=3-4 for each group). Different forms of 4EBP1, phosphorylated (p-4EBP1$^{T37/46}$ and p-4EBP1^{S65}), non-phosphorylated (non-p-4EBP1^{T46}) and total were analyzed. Similar to what was found after 11 i.v. administrations of AT-GAA, no changes in the mTOR activity are detected in treated compared to untreated KO samples. Again, similar to what was found after 11 i.v. administrations of AT-GAA, a decrease in both non-p-4EBP1^{T46} and total is seen in treated compared to untreated KO muscle GAPDH and vinculin were used as loading controls. Each data point represents an individual mouse. Statistical significance was determined by one-way ANOVA. Graphs represent mean ± SD. *P<0.05; ** P<0.01; *** P<0.001.

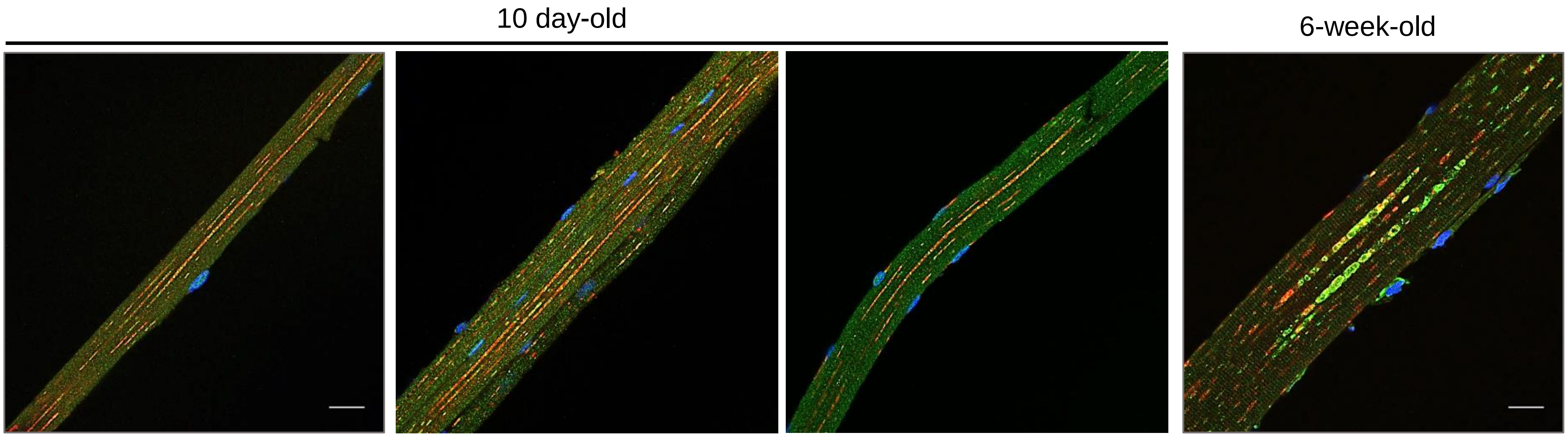

Figure S3. Autophagic buildup in KO muscle can be detected as early as in 10-day-old animals. Confocal microscopy images of single muscle fibers immunostained with lysosomal marker LAMP1 (red) and autophagosomal marker LC3 (green); nuclei are stained with Hoechst dye (blue). The fibers are isolated from muscle (the white part of gastrocnemius) derived from 10-day-old and 6-week-old mice. Bar: 20 μm.

Video S1. Confocal microscopy images of serial optical sections taken from two adjacent KO muscle fibers stained with lysosomal marker LAMP1 (red)and autophagosomal marker LC3 (green). The images show the extent of the autophagic buildup in fibers derived from untreated KO mice. Note, that the buildup in the left fiber surfaces before that in the right fiber, indicating that these structures belong to different focal planes along the Z stack. It is therefore clear that the autophagic buildup can be easily missed on routine histology of longitudinal sections of the diseased skeletal muscle.

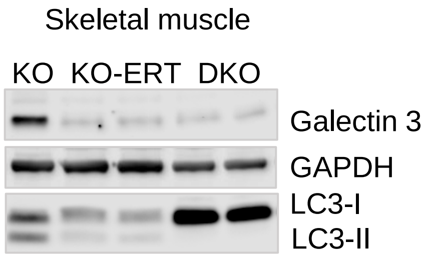

Raw data for Figure 5C

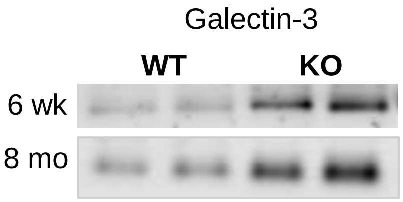

(referred to as “not shown” in the main text)

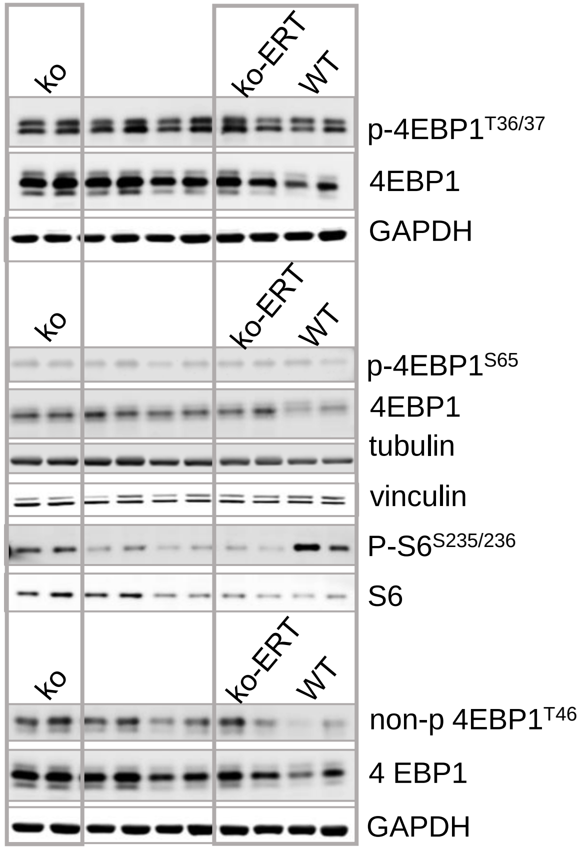

Raw data for Supplemental Figure S2

Supplemental Table 1: Absolute Concentration of 116 Compounds

ID	Compound name	KO-Treatment		KO		WT		KO-Treatment vs KO			KO vs WT		
HMT DB		Concentration (nmol/g)						Comparative Analysis					
		Mean	S.D.	Mean	S.D.	Mean	S.D.	Ratio ¶	*p*-value ‖		Ratio ¶	*p*-value ‖	
A_0001	NAD^+	479	74	548	19	485	61	0.9	0.071		1.1	0.052	
A_0002	cAMP	0.9	0.3	1.0	0.2	0.9	0.2	0.9	0.676		1.1	0.621	
A_0003	cGMP	N.A.	N.A.	0.02	0.002	N.A.	N.A.	<1	N.A.		1<	N.A.	
A_0004	NADH	9.6	0.7	11	1.5	9.4	1.0	0.9	0.223		1.1	0.147	
A_0005	Xanthine	1.1	0.3	1.8	1.0	0.4	0.3	0.6	0.159		4.2	0.021	*
A_0006	ADP-ribose	8.1	5.9	8.9	2.6	5.9	2.5	0.9	0.780		1.5	0.069	
A_0007	Mevalonic acid	N.A.	N.A.	N.A.	N.A.	N.A.	N.A.	N.A.	N.A.		N.A.	N.A.	
A_0008	UDP-glucose	18	5.7	24	1.2	15	6.6	0.8	0.052		1.6	0.023	*
A_0009	Uric acid	2.9	0.7	4.7	1.8	2.5	1.2	0.6	0.065		1.9	0.036	*
A_0010	$NADP^+$	12	2.1	17	1.3	11	1.3	0.7	0.001	**	1.6	7.0E-06	***
A_0011	IMP	78	20	98	32	158	79	0.8	0.235		0.6	0.131	
A_0012	Sedoheptulose 7-phosphate	N.A.	N.A.	N.A.	N.A.	N.A.	N.A.	N.A.	N.A.		N.A.	N.A.	
A_0013	Glucose 6-phosphate	1,427	372	1,343	217	1,765	271	1.1	0.643		0.8	0.015	*
A_0014	Fructose 6-phosphate	623	157	528	93	777	106	1.2	0.241		0.7	0.002	**
A_0015	Fructose 1-phosphate	N.A.	N.A.	N.A.	N.A.	N.A.	N.A.	N.A.	N.A.		N.A.	N.A.	
A_0016	Galactose 1-phosphate	5.6	1.3	8.2	2.7	2.9	1.1	0.7	0.070		2.8	0.003	**
A_0017	Glucose 1-phosphate	166	70	146	27	228	54	1.1	0.526		0.6	0.011	*
A_0018	Acetoacetyl CoA	N.A.	N.A.	N.A.	N.A.	N.A.	N.A.	N.A.	N.A.		N.A.	N.A.	
A_0019	Acetyl CoA	0.4	0.2	0.6	0.2	0.4	0.14	0.7	0.085		1.7	0.033	*
A_0020	Folic acid	N.A.	N.A.	N.A.	N.A.	N.A.	N.A.	N.A.	N.A.		N.A.	N.A.	
A_0021	Ribose 5-phosphate	2.6	1.7	4.3	1.7	2.4	0.9	0.6	0.109		1.8	0.041	*
A_0022	CoA	1.8	0.7	2.2	0.6	2.7	1.2	0.8	0.305		0.8	0.351	
A_0023	Ribose 1-phosphate	4.7	0.9	7.6	2.5	3.5	2.1	0.6	0.033	*	2.2	0.012	*
A_0024	Ribulose 5-phosphate	8.3	5.3	10	2.1	7.7	5.4	0.8	0.439		1.3	0.318	
A_0025	Xylulose 5-phosphate	N.A.	N.A.	N.A.	N.A.	N.A.	N.A.	N.A.	N.A.		N.A.	N.A.	
A_0026	Erythrose 4-phosphate	N.A.	N.A.	N.A.	N.A.	N.A.	N.A.	N.A.	N.A.		N.A.	N.A.	
A_0027	HMG CoA	0.3	0.008	0.4	0.04	0.3	0.006	0.9	0.308		1.1	0.615	
A_0028	Glyceraldehyde 3-phosphate	4.2	3.5	13	7.6	5.2	6.7	0.3	0.038	*	2.5	0.089	
A_0029	NADPH	5.8	0.2	5.9	0.4	6.3	0.3	1.0	0.611		0.9	0.069	
A_0030	Malonyl CoA	0.5	0.2	0.7	0.13	0.5	0.05	0.7	0.102		1.4	0.012	*
A_0031	Phosphocreatine	2,234	331	2,182	142	1,997	361	1.0	0.738		1.1	0.282	
A_0032	XMP	0.12	0.02	0.3	0.04	0.11	0.02	0.4	3.4E-05	***	3.0	1.7E-05	***
A_0033	Dihydroxyacetone phosphate	153	68	254	67	246	109	0.6	0.027	*	1.0	0.877	
A_0034	Adenylosuccinic acid	1.9	0.4	2.8	1.1	2.2	1.2	0.7	0.113		1.2	0.429	
A_0035	Fructose 1,6-diphosphate	353	205	469	110	635	482	0.8	0.258		0.7	0.443	
A_0036	6-Phosphogluconic acid	2.4	0.5	2.9	0.4	3.6	1.7	0.8	0.093		0.8	0.387	
A_0037	*N*-Carbamoylaspartic acid	0.07	0.03	0.11	0.03	0.10	0.04	0.6	0.018	*	1.2	0.451	
A_0038	PRPP	17	5.0	19	6.7	23	9.4	0.9	0.631		0.8	0.351	
A_0039	2-Phosphoglyceric acid	3.3	0.8	3.0	0.6	2.9	0.9	1.1	0.510		1.0	0.851	
A_0040	2,3-Diphosphoglyceric acid	1.1	0.2	2.2	1.0	0.8	0.07	0.5	0.033	*	2.7	0.018	*
A_0041	3-Phosphoglyceric acid	33	5.7	30	5.9	29	6.7	1.1	0.293		1.0	0.760	
A_0042	Phosphoenolpyruvic acid	6.9	1.0	2.8	1.6	4.4	1.3	2.5	7.1E-04	***	0.6	0.083	
A_0043	GMP	1.6	0.3	2.3	0.14	1.5	0.4	0.7	0.001	**	1.6	0.003	**
A_0044	AMP	4.9	1.2	5.8	2.6	4.3	2.2	0.8	0.463		1.4	0.294	
A_0045	2-Oxoisovaleric acid	4.9	1.2	5.0	0.5	7.7	2.1	1.0	0.860		0.7	0.045	*
A_0046	GDP	3.9	0.7	4.0	0.8	2.8	0.7	1.0	0.802		1.4	0.023	*
A_0047	Lactic acid	8,907	2,165	7,436	1,410	10,282	2,374	1.2	0.198		0.7	0.035	*
A_0048	ADP	187	106	144	56	124	63	1.3	0.400		1.2	0.572	
A_0049	GTP	128	6.9	151	3.5	111	6.1	0.8	1.0E-04	***	1.4	6.2E-07	***
A_0050	Glyoxylate	N.A.	N.A.	N.A.	N.A.	N.A.	N.A.	N.A.	N.A.		N.A.	N.A.	
A_0051	ATP	7,934	249	7,167	188	8,122	216	1.1	1.7E-04	***	0.9	1.1E-05	***
A_0052	Glycerol 3-phosphate	319	108	336	71	391	98	0.9	0.746		0.9	0.293	
A_0053	Glycolic acid	N.A.	N.A.	N.A.	N.A.	47	8.2	N.A.	N.A.		<1	N.A.	
A_0054	Pyruvic acid	55	18	45	17	63	28	1.2	0.354		0.7	0.208	
A_0055	*N*-Acetylglutamic acid	2.6	0.04	2.8	0.2	2.7	0.3	1.0	0.196		1.0	0.499	
A_0056	2-Hydroxyglutaric acid	3.6	1.0	5.3	1.2	20	2.0	0.7	0.026	*	0.3	3.3E-07	***
A_0057	Carbamoylphosphate	N.A.	N.A.	N.A.	N.A.	N.A.	N.A.	N.A.	N.A.		N.A.	N.A.	
A_0058	Succinic acid	157	49	232	82	142	42	0.7	0.093		1.6	0.046	*
A_0059	Malic acid	168	59	241	99	175	51	0.7	0.161		1.4	0.188	
A_0060	2-Oxoglutaric acid	N.A.	N.A.	N.A.	N.A.	N.A.	N.A.	N.A.	N.A.		N.A.	N.A.	

ID consists of analysis mode and number. 'C' and 'A' showed cation and anion modes, respectively.
N.D. (Not Detected): The target peak or metabolite was below detection limits.
N.A. (Not Available): The calculation was impossible because of insufficience of the data.
¶ The ratio is of computed by using averaged detection values. The latter was used as denominator.
‖ The p-value is computed by Welch's t-test. (*<0.05, **<0.01, ***<0.001)
The data are sorted by ID in ascending order.

Supplemental Table 1: Absolute Concentration of 116 Compounds

ID	HMT DB	Concentration (nmol/g)						Comparative Analysis					
	Compound name	KO-Treatment		KO		WT		KO-Treatment vs KO			KO vs WT		
		Mean	S.D.	Mean	S.D.	Mean	S.D.	Ratio ¶	*p*-value ‖		Ratio ¶	*p*-value ‖	
A_0061	Fumaric acid	62	17	106	31	59	15	0.6	0.018	*	1.8	0.013	*
A_0062	Citric acid	159	21	233	26	161	18	0.7	3.4E-04	***	1.4	3.8E-04	***
A_0063	*cis*-Aconitic acid	1.5	0.4	2.2	0.5	1.5	0.2	0.7	0.020	*	1.5	0.016	*
A_0064	Isocitric acid	N.A.	N.A.	N.A.	N.A.	N.A.	N.A.	N.A.	N.A.		N.A.	N.A.	
C_0001	Urea	5,551	1,006	4,799	857	6,610	1,046	1.2	0.194		0.7	0.009	**
C_0002	Gly	1,601	98	1,502	323	2,142	249	1.1	0.501		0.7	0.004	**
C_0003	Putrescine	0.2	0.3	1.0	0.4	0.9	0.4	0.2	0.080		1.1	0.686	
C_0004	β-Ala	13	1.4	14	1.2	27	2.6	1.0	0.490		0.5	1.1E-05	***
C_0005	Sarcosine	12	3.3	13	3.5	13	2.0	0.9	0.836		1.0	0.978	
C_0006	Ala	2,150	323	2,119	349	1,397	256	1.0	0.875		1.5	0.003	**
C_0007	γ-Aminobutyric acid	N.A.	N.A.	N.A.	N.A.	N.A.	N.A.	N.A.	N.A.		N.A.	N.A.	
C_0008	*N*,*N*-Dimethylglycine	4.8	1.0	5.4	1.4	4.6	0.6	0.9	0.536		1.2	0.409	
C_0009	Choline	41	23	23	17	N.A.	N.A.	1.8	0.214		1<	N.A.	
C_0010	Ser	275	25	252	51	224	28	1.1	0.353		1.1	0.277	
C_0011	Carnosine	616	62	529	14	1,859	90	1.2	0.017	*	0.3	1.9E-07	***
C_0012	Creatinine	59	9.6	45	4.2	58	2.8	1.3	0.012	*	0.8	1.6E-04	***
C_0013	Pro	86	29	57	24	59	14	1.5	0.103		1.0	0.898	
C_0014	Betaine	34	6.8	31	25	11	7.9	1.1	0.744		2.9	0.109	
C_0015	Val	205	49	167	35	186	28	1.2	0.153		0.9	0.300	
C_0016	Thr	219	37	208	37	192	27	1.1	0.590		1.1	0.440	
C_0017	Homoserine	N.A.	N.A.	N.A.	N.A.	N.A.	N.A.	N.A.	N.A.		N.A.	N.A.	
C_0018	Betaine aldehyde	N.A.	N.A.	N.A.	N.A.	N.A.	N.A.	N.A.	N.A.		N.A.	N.A.	
C_0019	Cys	N.A.	N.A.	N.A.	N.A.	N.A.	N.A.	N.A.	N.A.		N.A.	N.A.	
C_0020	Hydroxyproline	88	23	35	15	165	23	2.5	0.001	**	0.2	1.8E-06	***
C_0021	Creatine	16,237	1,323	16,165	1,010	16,769	2,025	1.0	0.919		1.0	0.534	
C_0022	Leu	149	41	136	33	105	15	1.1	0.565		1.3	0.079	
C_0023	Ile	95	23	78	19	73	11	1.2	0.207		1.1	0.583	
C_0024	Asn	65	18	76	22	45	5.6	0.9	0.373		1.7	0.018	*
C_0025	Ornithine	37	5.2	31	6.2	26	2.9	1.2	0.085		1.2	0.106	
C_0026	Asp	368	61	437	135	223	34	0.8	0.288		2.0	0.010	*
C_0027	Homocysteine	N.A.	N.A.	N.A.	N.A.	N.A.	N.A.	N.A.	N.A.		N.A.	N.A.	
C_0028	Adenine	3.1	0.5	3.4	0.8	3.0	0.3	0.9	0.551		1.1	0.331	
C_0029	Hypoxanthine	N.A.	N.A.	9.9	N.A.	N.A.	N.A.	<1	N.A.		1<	N.A.	
C_0030	Spermidine	1.1	0.3	2.2	0.8	2.6	0.7	0.5	0.024	*	0.8	0.356	
C_0031	Gln	2,036	95	2,289	260	1,742	232	0.9	0.064		1.3	0.003	**
C_0032	Lys	1,458	163	1,601	537	596	172	0.9	0.555		2.7	0.005	**
C_0033	Glu	767	241	1,041	290	726	182	0.7	0.106		1.4	0.053	
C_0034	Met	57	4.4	52	9.6	56	7.7	1.1	0.314		0.9	0.445	
C_0035	Guanine	N.A.	N.A.	N.A.	N.A.	N.A.	N.A.	N.A.	N.A.		N.A.	N.A.	
C_0036	His	189	22	173	22	140	13	1.1	0.227		1.2	0.011	*
C_0037	Carnitine	415	28	419	49	292	29	1.0	0.858		1.4	5.7E-04	***
C_0038	Phe	84	17	76	9.9	73	9.4	1.1	0.317		1.0	0.648	
C_0039	Arg	379	56	456	70	185	55	0.8	0.063		2.5	3.0E-05	***
C_0040	Citrulline	113	6.5	114	13	93	11	1.0	0.876		1.2	0.011	*
C_0041	Tyr	69	11	65	6.7	83	11	1.1	0.445		0.8	0.006	**
C_0042	*S*-Adenosylhomocysteine	4.2	0.3	4.2	0.4	4.6	0.2	1.0	0.882		0.9	0.207	
C_0043	Spermine	2.7	0.3	2.7	0.12	2.3	0.2	1.0	0.958		1.2	0.147	
C_0044	Trp	25	3.3	28	4.6	29	2.8	0.9	0.239		1.0	0.707	
C_0045	Cystathionine	N.A.	N.A.	N.A.	N.A.	N.A.	N.A.	N.A.	N.A.		N.A.	N.A.	
C_0046	Adenosine	9.3	3.7	11	4.2	6.1	1.8	0.8	0.434		1.8	0.031	*
C_0047	Inosine	14	2.9	27	7.5	13	2.5	0.5	0.006	**	2.1	0.004	**
C_0048	Guanosine	2.0	0.2	2.4	0.2	N.A.	N.A.	0.8	0.017	*	1<	N.A.	
C_0049	Argininosuccinic acid	5.1	0.6	6.7	1.2	3.8	0.2	0.8	0.020	*	1.8	0.002	**
C_0050	Glutathione (GSSG)	335	52	399	88	234	81	0.8	0.164		1.7	0.007	**
C_0051	Glutathione (GSH)	299	96	300	111	376	146	1.0	0.992		0.8	0.332	
C_0052	*S*-Adenosylmethionine	22	0.7	26	2.2	20	0.7	0.9	0.007	**	1.3	7.5E-04	***

ID consists of analysis mode and number. 'C' and 'A' showed cation and anion modes, respectively.
N.D. (Not Detected): The target peak or metabolite was below detection limits.
N.A. (Not Available): The calculation was impossible because of insufficience of the data.
¶ The ratio is of computed by using averaged detection values. The latter was used as denominator.
‖ The p-value is computed by Welch's t-test. (*<0.05, **<0.01, ***<0.001)
The data are sorted by ID in ascending order.
